# The clean energy claims of BP, Chevron, ExxonMobil and Shell: A mismatch between discourse, actions and investments

**DOI:** 10.1371/journal.pone.0263596

**Published:** 2022-02-16

**Authors:** Mei Li, Gregory Trencher, Jusen Asuka

**Affiliations:** 1 Graduate School of Environmental Studies, Tohoku University, Sendai, Miyagi Prefecture, Japan; 2 Graduate School of Global Environmental Studies, Kyoto University, Kyoto, Japan; 3 Center for Northeast Asian Studies, Tohoku University, Sendai, Miyagi Prefecture, Japan; Texas A&M University, UNITED STATES

## Abstract

The energy products of oil and gas majors have contributed significantly to global greenhouse gas emissions (GHG) and planetary warming over the past century. Decarbonizing the global economy by mid-century to avoid dangerous climate change thus cannot occur without a profound transformation of their fossil fuel-based business models. Recently, several majors are increasingly discussing clean energy and climate change, pledging decarbonization strategies, and investing in alternative energies. Some even claim to be transforming into clean energy companies. Given a history of obstructive climate actions and “greenwashing”, there is a need to objectively evaluate current and historical decarbonization efforts and investment behavior. This study focuses on two American (Chevron, ExxonMobil) and two European majors (BP, Shell). Using data collected over 2009–2020, we comparatively examine the extent of decarbonization and clean energy transition activity from three perspectives: (1) keyword use in annual reports (*discourse*); (2) business strategies (*pledges and actions*); and (3) production, expenditures and earnings for fossil fuels along with investments in clean energy (*investments*). We found a strong increase in discourse related to “climate”, “low-carbon” and “transition”, especially by BP and Shell. Similarly, we observed increasing tendencies toward strategies related to decarbonization and clean energy. But these are dominated by pledges rather than concrete actions. Moreover, the financial analysis reveals a continuing business model dependence on fossil fuels along with insignificant and opaque spending on clean energy. We thus conclude that the transition to clean energy business models is not occurring, since the magnitude of investments and actions does not match discourse. Until actions and investment behavior are brought into alignment with discourse, accusations of greenwashing appear well-founded.

## 1. Introduction

Efforts to limit planetary warming to below 2°C or 1.5°C entail a transition to net-zero-emissions energy systems by 2050 [1]. This carries vast implications for fossil fuel producers. Twenty fossil fuel companies are responsible for 35% of all energy-related carbon dioxide (CO_2_) and methane emissions worldwide since 1965 [2, 3]. The leading investor-owned emitter is Chevron, followed closely by Exxon, BP, and Shell. The products of these four energy giants account for more than 10% of global carbon emissions since 1965 [2, 3].

As the world shifts away from fossil fuels to mitigate climate change and air pollution, oil majors’ historically prosperous business models must grapple with the likelihood of decreased demand for hydrocarbons and fewer opportunities for profit [4], especially since the pandemic situation [5]. Symbolically illustrating this, in 2020, ExxonMobil dropped from the Dow Jones Industrial Average Index after nearly a century [6]. Meanwhile, electric-vehicle maker Tesla’s market value reached US $800 billion, propelling CEO Elon Musk to the status of the world’s richest man [7]. BP even suggested that oil demand may already have peaked [8]. Multiple developments are driving this shift. These include the electrification of road transport and government policies aiming to phase out internal combustion engines [9], divestment targeting the fossil fuel industry [10], climate policies targeting fossil fuel extraction and usage [11, 12] as well as government-led lawsuits and inquiries (including the U.S. Congress) against oil companies for delaying climate action and disseminating misinformation [13–17]. To survive in this changing market, oil majors face an urgent need to transition to a carbon-free business model [18, 19]. Indeed, some suggest that to meet the Paris Agreement goals, no additional CO2-emitting fossil fuel infrastructure should be commissioned, and early retirement of existing projects is required [1].

Oil majors have made both proactive and regressive steps toward this. Recently, some have begun investing in renewables and low-carbon technology [18, 20]. Many have announced various targets to mitigate greenhouse gas (GHG) emissions from operations or products [21] while stating public support for carbon pricing [22, 23]. Some—such as BP and Repsol—even claim to be transitioning to clean energy [3].

However, historical behavior suggests that the authenticity of such claims should be examined critically and exhaustively. Multiple studies document how oil majors have strategically spread misinformation and aggressively obstructed progress toward climate action. ExxonMobil is a flagrant example, having strategically denied climate change and propagated disinformation to mislead the public for over 20 years [24–26]. Multiple majors have tried to shift the responsibility for climate change onto consumers (BP’s promotion of reducing individual carbon footprints is one example) [25, 27, 28]. The biggest American and European majors have also spent millions lobbying to delay or weaken climate policy [3, 15, 29]. Recently, oil majors are observed to be using advertising on social media platforms to influence public opinion and promote an image of green fossil fuels [30]. Research [3, 31] finds that even the most ambitious majors are simply mitigating future carbon risks by diversifying energy products rather than pursuing company-wide decarbonization. Given this track record, decarbonization claims by oil majors have been critiqued as “greenwashing” [32–34]. This is where corporate strategies—especially discourse and pledges to stakeholders—depict proactive actions that exceed a company’s actual environmental performance [35, 36].

This situation points to a strong need to comprehensively investigate the actual state of actions by oil majors to decarbonize and transition to clean energy. Previous scholarship has tackled this from diverse but disparate perspectives. This includes societal accountability [22, 23], the spreading of climate disinformation [24], lobbying [29, 37], emissions reductions efforts for operations and downstream products [38, 39] and renewables investment [21, 40]. Some recent studies focus on broader efforts to transition business models and phase out fossil fuel production [41].

This literature has limitations. Typically focusing on a single year [21, 22, 38, 42], prevalent approaches cannot fully identify the evolving nature of decarbonization trajectories. A long-term perspective is critical, since fossil fuel majors differ from smaller entities, whose transition strategies might proceed faster. Additionally, most research focuses on public statements or a narrow range of business strategies [22]. This hampers the ability to comprehensively monitor the complex and intertwined strategies making up a clean energy transition. Relying only on public statements alone also risks overestimating a company’s performance due to greenwashing tactics. Thus, objectively examining the extent of transition strategies by oil majors requires a multi-year approach, combining quantitative and qualitative data.

This study, therefore, assesses the extent to which oil majors are divesting from fossil fuels and transitioning toward clean energy. Focusing on four—ExxonMobil, BP, Shell, and Chevron—we examine behavior from three perspectives: (1) *discourse*: frequency of climate- and clean-energy-related keyword use in annual reports; (2) *strategies*: pledges and actions related to decarbonization and clean energy and (3) *investments*: production, expenditures and earnings for fossil fuels as well as investments in clean energy. The analysis covers over a decade of activity (2009–2020), using publicly available qualitative and quantitative data.

Our scholarly contribution is two-fold. Empirically, we reveal the actual extent to which each major is moving toward a clean-energy-based business model. Theoretically, our analytical framework can be used or built on by scholars to measure the transition behavior of other fossil fuel-centric industries.

## 2. Methods

### 2.1 Sample selection

Four oil majors were selected for analysis: two from the US (Chevron, ExxonMobil) and two from Europe (BP, Shell). Together with Total, ConocoPhillips, and Eni, they comprise a larger group of seven international oil companies listed on the American and European stock markets [4]. We selected these four for two reasons. First, based on product-based GHG emissions since 1965, this group represents the top-ranking emitters among all investor-owned energy majors. Second, the balance of two American and two European majors allows analysis of contrasting attitudes and responses to the energy transition. Specifically, prior research [31, 41–43] has shown the American majors as resistant or obstructive, and the European companies as slightly more proactive.

### 2.2 Study design and methods

The aim of this study is to assess the extent to which the above four oil majors are transitioning from a business model based primarily on fossil fuels towards one based on clean energy. In line with literature [44, 45], we expect this transition to involve a continual and multi-year process whereby oil and gas businesses are gradually contracted while investments in renewables are expanded, eventually replacing fossil fuel extraction and sales. We also expect the ultimate goal of such a transition to be net-zero; to eliminate all GHG emissions that result from the production or use of energy products [4, 46]. To measure and compare each major’s transition activities, in accord with our triple analysis of *discourse*, *strategies* and *investments*, our study design consists of the following three steps and sets of assumptions. Data for all steps covers the 12-year study period 2009–2020.

#### 2.2.1 Discourse

The first step examines the frequency of 39 keywords in annual reports published during the study period (Table 1). Following literature [47], we counted the frequency of each term and variants, dividing by the total word count in each report to normalize results. Terms were identified first from oil-industry grey literature [4, 48, 49] then organized into four categories: (i) *climate change*, focused on detecting awareness of climate-related concepts; (ii) *transition*, examining discourse reflecting a resolve to transition business models; (iii) *emissions*, showing acknowledgment of the need to reduce various GHG emissions; and (iv) *clean energy*, reflecting statements related to investments in non-fossil fuel or decarbonized energies. These categories capture the cognition of four key perspectives deemed essential to the clean-energy transformation of the oil industry.

**Table 1:** Keywords examined in the discourse analysis.

Climate change		Transition		Emissions		Clean energy	
Main terms	Variants	Main terms	Variants	Main terms	Variants	Main terms	Variants
1.5 degree*	1.5°C	carbon + variant	activit*;business*; credit*; cost*; offset*; polic*; pric*; project*; tax*	carbon + variant	abatement; dioxide*; CO2; emission*; footprint; intensity; neutral*; zero-, sink*	alga*	
2 degree*	2°C; two degree*	decarbon*		ccs/ccus	carbon capture; carbon storage; carbon removal	alternative + variant	fuel, energ*
climate		transition*		energy efficiency	energy efficient	batter*	
dual challenge		sustainab*		flar*		biofuel*	biopower, bioenerg*, biomass
OGCI	Oil and Gas Climate Initiative			fluorinat*		clean* + variant	fuel, energ*
IPCC	Intergovernmental Panel on Climate Change			greenhouse	GHG	electric vehicle	electric mobility; electric transport; EV, charging; charger*
Kyoto				Hydrofluorocarbon*	perfluorocarbon*	electricit*	power*
Paris				sulfur hexafluoride		ethanol	methanol
UNFCCC	United Nations Framework Convention on Climate Change			methane	CH4	geothermal	
warming				net zero	zero net	hydropower	
				N2O	nitrous oxide	hydrogen	
						low* carbon	
						renewable*	
						solar	
						wind	

Note: More comprehensive information on the omitted keywords appears in S1 File.

In selecting this approach, we assume that the frequency by which keywords are used will provide a rough proxy for the degree of awareness and importance placed on these issues [50]. Not overlooking the possibility of greenwashing [51], we view discourse and attention to climate and clean-energy issues as a precursor to concrete actions like investments and organizational transformation [52]. We focus on annual reports because they are the most official and representative of the various documents written to shareholders and stakeholders, and because their consistent year-to-year format is well suited to comparisons.

S1 File details our procedure for selecting and omitting keywords. S1 Dataset contains all data collected and processed for this step.

#### 2.2.2 Strategies: Pledges and actions

Our second step identifies and compares the annual status of strategies reflecting a transition towards a clean-energy business model. We sourced 25 indicators from academic and grey literature (Table 2) and organized these into four categories. These capture the presence of pledges and disclosure (14 indicators; shown as “P&D”) as well as concrete actions (11 indicators; shown as “A”). When referring to indicators, we use codes. For example, “BM1-P&D” refers to indicator 1 (pledges and disclosure) of the business model category.

**Table 2:** Indicator descriptions.

Category	Sub-category	Indicator	Type	Basis in literature
***Climate change cognition***	Awareness of climate change	CC1. Does the major acknowledge the scientific evidence of anthropogenic climate change (e.g. the link between human activities or fossil fuels and climate change, and potential risks or dangers of climate change etc.)?	P&D	[50, 52]
		CC2. Does the major affirm the need for itself or society to shift away from or reduce dependence on all types of non-sequestered fossil fuels to mitigate climate change?	A	
	Participation in international framework	CC3. Has the major joined the Oil and Gas Climate Initiative (OGCI)?	A	[31, 43]
	Disclosing climate risk	CC4. Does the major disclose regulatory risks related to climate change on their business or products?	P&D	[23]
		CC5. Does the major disclose market and other indirect risks and opportunities due to increasing climate concerns (e.g. reduction of market returns, shifts in consumer preferences, competition from renewables and transport electrification etc.)?	P&D	
***Business model***	Transition strategy	BM1. Has the major pledged to shift their assets and product portfolio to carbon-free energy in the long-term (in the next few decades)?	P&D	[19, 54, 59, 65]
		BM2. Has a step-by-step strategy been formulated to achieve this?	A	
	Fossil fuel production	BM3. Does the major pledge to reduce the production of all non-sequestered fossil fuels annually due to climate concerns?	P&D	[57, 58, 66]
		BM4. Has the major reduced the production volume of all non-sequestered fossil fuels in a given single or multi-year period due to climate concerns?	A	
	Fossil fuel exploration	BM5. Does the major pledge to reduce their exploration of fossil fuels due to climate concerns?	P&D	[41, 46, 55]
		BM6. Has the major reduced the exploration or estimates of fossil fuel reserves under holding because of climate concerns?	A	
	Workforce reallocation	BM7. Does the major pledge to reallocate the labor force to low-carbon businesses?	P&D	[51]
		BM8. Has a step-by-step strategy been formulated to achieve this?	A	
	Carbon price	BM9. Does the major state support for carbon pricing policies by governments (e.g., taxes or emissions trading, etc.) to mitigate climate change and promote clean energy?	P&D	[56, 64, 67, 68]
		BM10. Has the major employed a carbon price or tax into their internal investment decisions?	A	
***Emissions reduction***	Carbon emissions	ER1. Does the major pledge a long-term goal to reach net-zero carbon or GHG emissions on an absolute basis by the year 2050 or sooner, at least for scope 1 and scope 2 emissions?	P&D	[4, 21, 38, 40, 42, 46, 60, 67, 69]
		ER2. Has a concrete strategy been formulated to achieve this (e.g. an integrated series of steps or more specific targets)?	A	
	Scope 3 emissions	ER3. Does the major pledge to reduce scope 3 emissions?	P&D	[4, 21, 62, 63]
		ER4. Has a concrete strategy been formulated to achieve this (e.g. an integrated series of steps or more specific targets)?	A	
	Methane emissions	ER5. Does the major pledge to reduce methane emissions, on an absolute or intensity basis, for the following years?	P&D	[4, 48]
		ER6. Has a concrete strategy been formulated to achieve this (e.g. an integrated series of steps or more specific targets)?	A	
	Emissions disclosure	ER7. Does the major disclose all three scope GHG emissions annually?	P&D	[23, 41]
***Clean Energy***	Clean energy investment	CE1. Does the major publicly disclose the total annual investment volume in clean energy (e.g. clean fuels or electricity production, R&D, etc.)?	A	[31, 39, 70]
		CE2. Does the major pledge to allocate a specific portion (at least 1%) of their annual capex or investments to clean energy technologies (e.g. clean energy production, carbon capture and storage etc.)?	P&D	[4, 46, 60]
		CE3. Has the major allocated at least 1% of their annual capex or investments to clean energy technologies (e.g. clean energy production, carbon capture and storage etc.)?	A	

Note: P&D indicates pledges and disclosure; A indicates actions.

*2*.*2*.*2*.*1 Climate-change cognition (CC)*. Providing the intellectual justification to take action, these indicators serve as preconditions or predictors for pursuing a transition to clean energy [22]. The first two measure the presence of official statements acknowledging the link between human activities and climate change (CC1-P&D) and the need to reduce either emissions from fossil fuel combustion or production volumes (CC2-P&D). Next, participation in industry coalitions for decarbonization has incited pro-climate behavior amongst oil majors [31]. We thus measure participation in the Oil and Gas Climate Initiative (OGCI) (CC3-A) due to its status as the leading global framework for guiding the oil industry’s response to climate change [48, 53]. We also examine recognition of two risks related to climate change: regulatory and market (CC4-P&D, CC5-P&D).

*2*.*2*.*2*.*2 Business model (BM)*. This category measures the presence of a concrete transition strategy (BM1-P&D and BM2-A), along with pledges and actions to reduce exploration or production of non-sequestered fossil fuels due to climate concerns (BM3-P&D to BM6-A) and to transition the workforce to clean-energy businesses (BM7-P&D and BM8-A). We measure support for government carbon-pricing policies (BM9-P&D) and the introduction of carbon costs into internal decision-making (BM10-A). These strategies are widely recognized as indicating a shift toward clean energy [54–59].

*2*.*2*.*2*.*3 Emissions reduction (ER)*. This category measures the presence of pledges and actions to reduce GHG emissions. Since the concrete goal of achieving zero emissions by 2050 is shared globally [60, 61], we evaluate if each major pledges to reach net-zero on an absolute basis by 2050 or sooner (ER1-P&D, ER2-A). Observing the growing importance of reducing the GHG emissions resulting from end-user combustion of energy products [4, 62, 63], we evaluate the presence of pledges and strategies to reduce scope 3 emissions intensity (ER3-P&D, ER4-A). We also search for multi-year ambitions and strategies to reduce methane emissions (ER5-P&D, ER6-A), and complete disclosure of all GHG emissions for scope 1, 2, and 3 (ER7-P&D).

In designing these indicators, we exclude targets to reduce GHG emissions and actual emissions performance each year. This reflects the view that relative emissions per unit of output are naturally decreasing due to technical improvements in operation [3,43], and that absolute emissions each year are principally determined by hydrocarbon production volumes. Thus, focusing on these annually declining emissions metrics would not reveal behavior consistent with a transition to clean energy.

*2*.*2*.*2*.*4 Clean energy investment (CE)*. This category emphasizes investments in renewables—the core driver of transition [64]. We measure whether each major transparently discloses the annual volume of investments made in clean energy (CE1-A), and whether this is above 1% of total CAPEX (CE2-P&D, CE3-A). This highly conservative 1% threshold follows observations that investment volumes by oil majors, being still outside their core business areas, have been consistently less than this amount [4].

Data to apply this framework was sourced from annual reports, sustainability reports, and energy-transition reports published by each major during the study period. Based on quantitative or collective evidence, we evaluated each major’s actions using numerical scoring:

- “+1” for pledges and actions that implement or reinforce a strategy or commitment in that year. Taking indicator BM1-P&D for example (which measures the presence of a pledge to shift assets and product portfolio to clean energy), if a major fixed the goal to reach net-zero emissions by 2050, a score of “+1” would be allocated to all years this pledge was observed or remained active.
- “-1” for pledges and actions that contradict or hamper a strategy or commitment in that year. Taking indicator BM3-P&D for example (which assesses whether a company pledges to reduce annual production of non-sequestered fossil fuels due to climate concerns), if we found a statement about increasing fossil fuel production, this contradicts attainment of the indicator. Hence, a score of “-1” would be given in that year.
- “0” when no evidence was found of pledges and actions in either direction. This also includes strategies that, although possibly implemented or continuing from previous years, are not mentioned in the studied documents.

We provide concrete examples of scoring decisions for all indicators in (S2 File).

Additionally, all data collected and examined for this step is available in (S2–S5 Datasets).

#### 2.2.3 Investment: Production, earnings and expenditures

The third step analyzes financial-performance data collected from each major’s annual reports over the study period. This tracks changes in annual financial activity in six areas: (i) CAPEX for upstream businesses related to fossil fuels (i.e. oil and gas), which includes exploration, field development and production, midstream transportation, storage and processing, and the marketing and trading of oil and natural gas, (ii) associated earnings, (iii) fossil fuel production volumes, (iv) fossil fuel reserve estimates, (v) downstream sales, and (xi) CAPEX in clean-energy production and technologies. In processing data, we mainly use ratios instead of absolute volumes. Relative amounts (i.e. ratios) are better suited to showing the structure of each major’s earnings and production portfolio, since absolute volumes (e.g. CAPEX, fossil fuels sales) vary significantly each year with changing market situations (e.g. oil prices).

First, analysis of CAPEX volumes shows each major’s annual spending—through acquisition, upgrading, and maintenance—on fixed assets supporting the upstream production of oil and gas (original data is reported as millions of dollars). We calculate the percentage of fossil fuel-related CAPEX relative to total CAPEX, which also includes downstream and other capital outlays. Collection of this data is based on expectations that a major undergoing transition would reduce CAPEX volumes for upstream fossil fuel production, channeling investments toward clean-energy businesses [71].

Second, analysis of total annual fossil fuel earnings reveals the business model’s dependence on fossil fuels. We report absolute earnings in millions of dollars first, then convert each to a relative share of total earnings. Again, we assume that both absolute and relative volumes of upstream earnings from fossil fuels would decline during a clean-energy transition. We expect this to occur as each major achieves a greater relative share of earnings from clean energy instead of fossil fuels [72]. Conversely, we expect that a higher dependence on fossil fuels for total earnings would reduce the economic rationale to transition [73, 74].

Third, analysis of average daily production volumes for oil (i.e. liquids) and gas is firstly shown as units of one thousand barrels per day for liquids, and millions of cubic feet per day for gas. In addition to showing combined annual production of oil and gas, we convert each to an incremental percentage, setting 2009 as the baseline. This allows us to minimize the influence of fossil fuel price fluctuation, since we focus on production volumes instead of sale revenues. Underpinning this analysis is the logic that curbing the *supply* of fossil fuels is crucial for meeting the temperature targets of the Paris Agreement [66, 75]; and that majors might choose to increase gas production due to its lower carbon intensity than oil [76].

Fourth, analysis of each major’s annual estimates of oil and gas reserves is based on original data reported as millions of barrels for liquids, and billions of cubic feet for gas. We again change to an incremental percentage, setting 2009 volumes as the baseline. In including this dimension, we expect those majors with greater fossil fuel reserves to experience the largest difficulties with leaving these assets in the ground, because of their financial value to share prices and future production [77–79].

Fifth, analysis of annual downstream petroleum sales is based on original data reported as one thousand barrels per day for refined oil, and one thousand tons per day for chemicals. We focus again on sales volumes of refined oil and chemicals, converting data to an incremental ratio, setting 2009 as the baseline. Here we assume that the reduced sale of processed hydrocarbon products, being a principal source of scope 3 emissions alongside unrefined oil and gas, would be another important goal for transitioning to a clean-energy-based business model [80].

Sixth, we compare two types of data for renewable energy investments: (i) expenditures in renewables production and technology development, shown as a share of total CAPEX; and (ii) renewable electricity generation capacity, shown as megawatts (MW). Data is not fully disclosed in annual reports, obliging us to use third-party sources, whose data shows only cumulative rather than annual trends. Specifically, CAPEX data sourced from CDP (2019) and Fletcher et al. (2018) [67, 81] shows only cumulative investments from 2010 to the third quarter of 2018. Meanwhile, data on renewable-electricity capacity, sourced from the Standard & Poor’s Global Platts website and database [82], shows only total amounts from 2009–2019.

All data collected and processed for this step is available in (S6 Dataset).

## 3. Findings

### 3.1 Discourse

**Fig 1:**
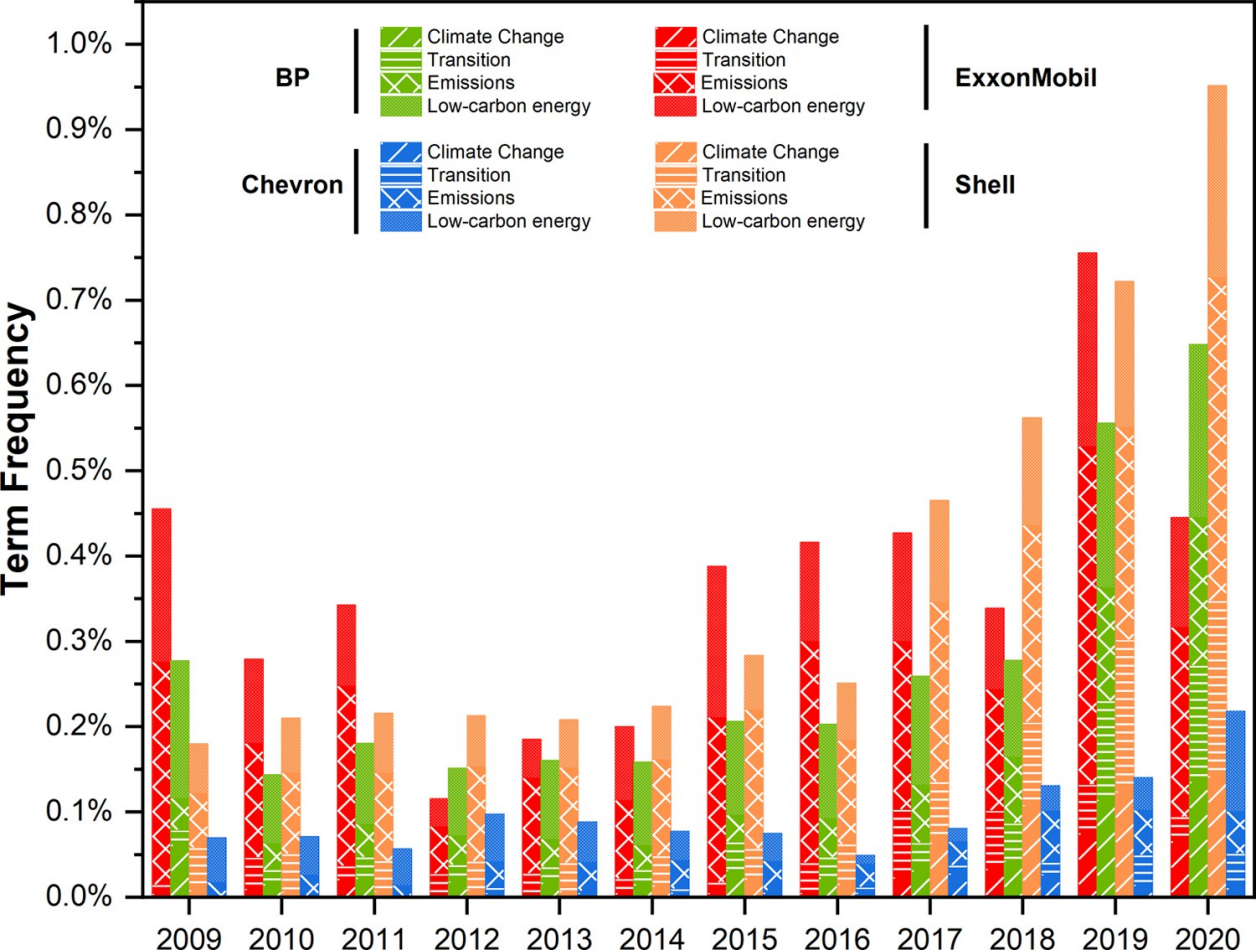
Frequency of keyword mentions in annual reports (normalized by total word count). Note: Results for ExxonMobil are affected by the style of reports examined (all years except 2020 are summary reports).

Fig 1 shows the normalized results of the discourse analysis: the frequency of 39 keywords in annual reports. All majors show a clear increasing trend over the study period, most notably the European majors, particularly in the “transition” and “emissions” categories. Findings reflect an amplification of discourse about mitigating GHG emissions and increasing clean energy businesses.

Shell is the only company showing a marked increase in all four categories over the study period, displaying a clean “J” curve. Notable keywords include “low-carbon energy” (increasing almost ten-fold, from 59 to 503 mentions), “renewable” (3 to 91), and “clean” (9 to 82). This shift in oral discourse is also visible in the evolving messages from Shell’s chairpersons. For example, in 2009, Jorma Ollila stated ambitions to produce more oil and natural gas to meet global energy demands [83, 84]. This was flipped in 2020, as Chad Holliday pledged a reoriented mission, to “play an essential role in the move to a cleaner, lower-carbon world” [83].

BP also shows a marked increase in keyword usage over the study period, especially in 2019. In contrast to Shell’s steadily rising trend, BP’s follows a “U” curve, with low points between 2012 and 2017. BP has increased usage of words in the “climate change” category in particular, from 22 to 326 mentions over 2009–2020. The “transition” category also increased markedly in the study period, from 50 to 418, reflecting increased discussion of a low-carbon business model. In 2009 the CEO considered BP an “oil company”, whereas in 2021, BP advocated transforming into an “integrated energy company” and pledged a transition to net-zero emissions [84].

Chevron is the only major not showing a noticeable increase. It trails the European majors in all categories, particularly “climate” and “transition”. The word “climate” was mentioned only 45 times for the entire study period and was missing from annual reports in 2009–2011. This compares to 171 mentions by BP in 2020 alone. Chevron’s lagging performance is also indicated by mention of “renewables”. This appears only in the glossary of terms of the 2009 annual report, and climbs to just 19 mentions in 2019 [85], compared to 92 times by BP.

Interpreting ExxonMobil’s results requires caution. This major increased its usage of keywords after 2014. But this is likely influenced by the style of annual reports. While the other three majors release full reports each year, ExxonMobil published only summaries (except 2020). Considering that total normalized keyword usage dips markedly in 2020 (when a full report was available), for other years, results appear to be inflated by the brevity of annual-report summaries. This aside, ExxonMobil’s results show an increase in discourse on “emissions” and “low carbon” energy. But the low frequency of keyword mentions in the “climate change” and “transition” categories reflects low attention to these issues. This differs from the European majors, where results in the “transition” category increase markedly. Furthermore, ExxonMobil’s use of several keywords is erratic. For example, in the “emissions” category, although “CO2” was mentioned 10 times in 2009 and 11 times in 2020, it was not found between 2011 and 2013.

### 3.2 Strategies: Pledges and actions

**Fig 2:**
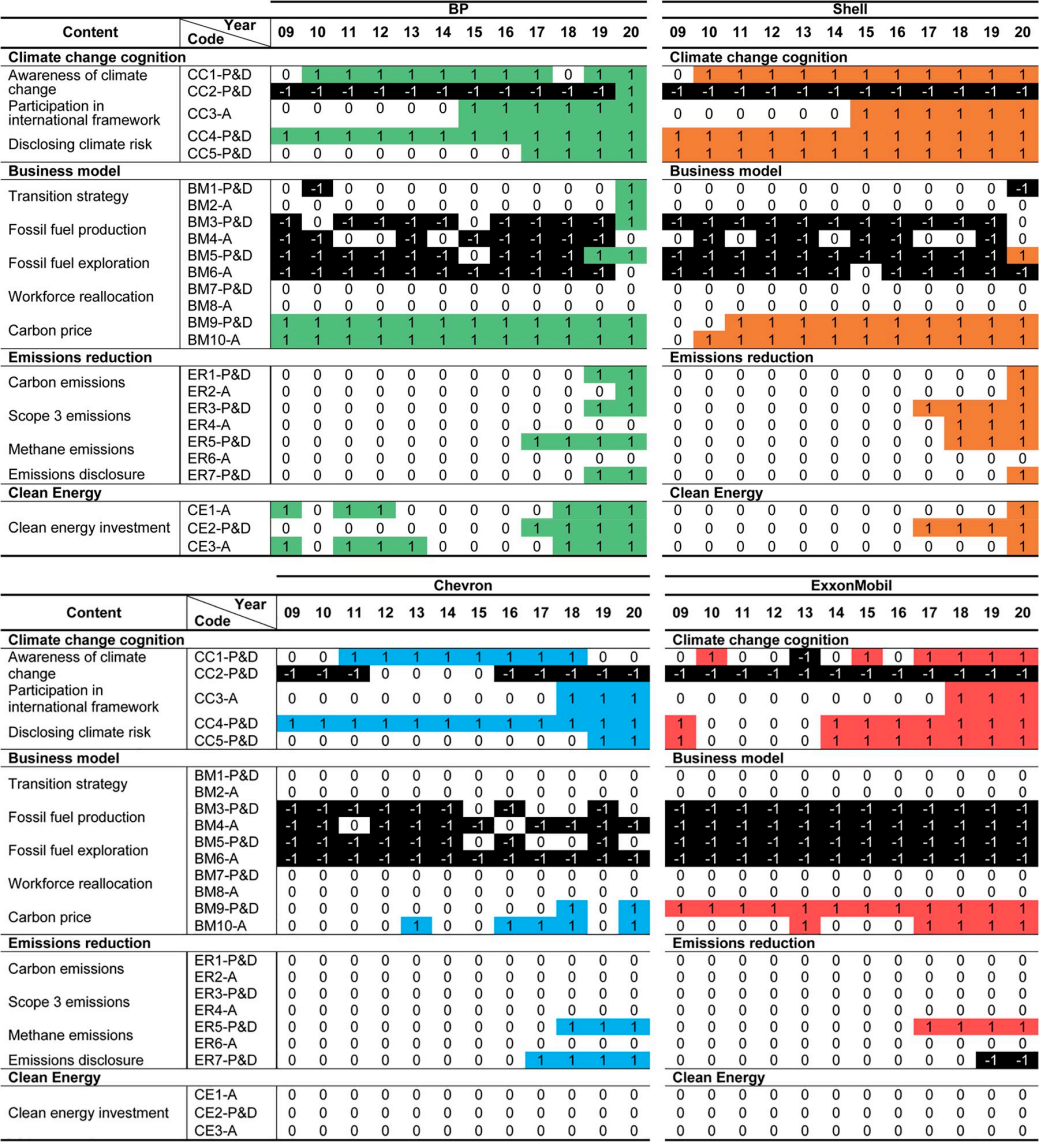
Business strategy analysis for European majors (2009–2020). (a) Business strategy analysis for European majors. (b) Business strategy analysis for American majors. Note on scores: “+1” indicates pledges and actions that implement or reinforce a strategy or commitment in that year; “-1” indicates pledges and actions that contradict or hamper a strategy or commitment in that year; and “0” indicates that no evidence of pledges and actions in either direction was found.

**Fig 3:**
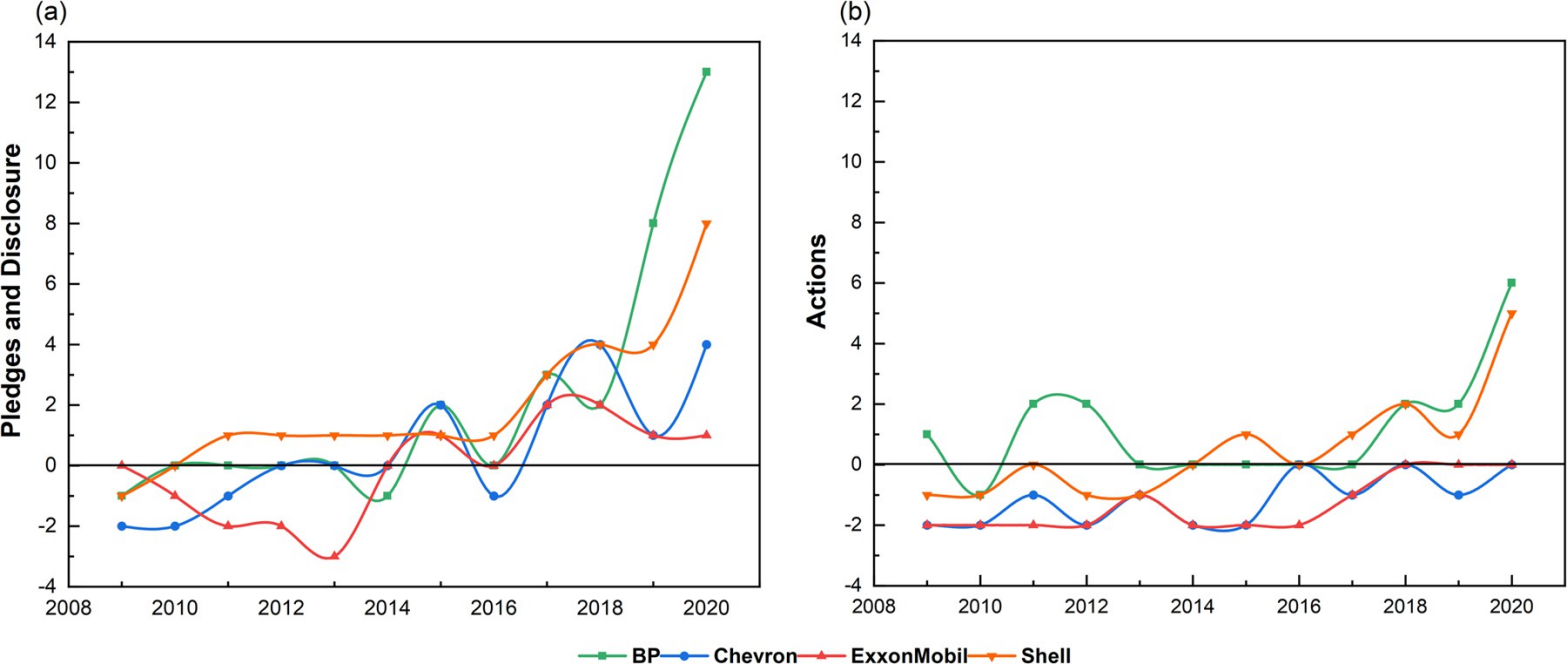
Total annual scores for all business strategies (2009–2020). (a) Total scores for pledges (b) Total scores for actions. Note: Total possible scores each year for pledges and actions are 14 and 11, respectively (1 for each indicator).

This section applies the framework explained in Section 2.2.3 and Table 2 to evaluate the state of pledges and concrete business actions that indicate a shift to clean energy. Results for each major appear in Fig 2A and 2B. The differing trajectories of pledges and actions appear in Fig 3. Detailed supporting evidence is provided in (S2–S5 Datasets). When discussing findings, we refer to indicators with codes explained in the Methods (Section 2.2.2).

#### 3.2.1 Climate-change cognition

While the two European majors consistently acknowledge the anthropogenic causes of climate change (CC1-P&D) over the study period, the American majors have frequently ignored this topic. Only in 2018 did ExxonMobil recognize, indirectly and weakly, the link between fossil fuels and climate change in its annual report—and this position did not carry over into the 2020 version. Meanwhile, from 2011 to 2017, Chevron conceded in its corporate responsibility report that “use of fossil fuels to meet the world’s energy needs contributes to the rising concentration of greenhouse gases in Earth’s atmosphere” [86]. Yet this acknowledgment vanished from later versions.

In contrast, the European majors have consistently acknowledged the link between fossil fuels and climate change. For Shell, this began in 2010 and evolved into stronger annual-report statements from 2015 on, such as: “We have long recognized that the use of fossil fuels contributes to climate change” [87]. BP follows a similar trend: its acknowledgment of climate change begins weakly in sustainability reports from 2010 [88], and evolves into stronger statements from 2012 on, such as: “action is needed to limit carbon dioxide (CO_2_) and other greenhouse gases being emitted through fossil fuel use” [89].

For both the American and European majors, we find scant acknowledgment of the need to shift away from or reduce dependence on all types of non-sequestered fossil fuels (CC2-P&D). Overall, statements consistently argue the reverse. For example, from 2014, ExxonMobil began to reject the need to reduce emissions from hydrocarbon development [90], even strengthening this position in 2020: “With respect to energy supply, production reductions by individual companies would have no impact on demand or consumption of energy, and would simply result in production shifting from one producer to another” [91]. Chevron expresses similar views, stating as recently as 2019 that “a decrease in overall fossil fuel emissions is not inconsistent with continued or increased fossil fuel production by the most efficient producers” [92]. The European majors show a similar pattern of rejecting the need to transition away from non-sequestered fossil fuels. BP communicated in its annual report 2019 that producing more gas was consistent with a net-zero future [8]. This only changed in 2020, when BP announced the intention to reduce all fossil fuel production as part of its energy transition [84].

As the only indicator for action in this category, participation in the Oil and Gas Climate Initiative (CC3-A), established in 2014, indicates more rapid acknowledgment by the European majors of the need to begin the energy transition. Specifically, BP and Shell joined in 2015, three years before ExxonMobil and Chevron.

Finally, BP, Shell, and Chevron have actively disclosed climate-related regulatory risk since 2009 (CC4-P&D). Shell was the first to consistently acknowledge both regulatory and market risks from climate change in its annual reports. For the other three, disclosure of market risk (CC5-P&D) has probably lagged due to views that renewables would not pose a significant threat to hydrocarbon businesses in the short term [90, 93].

#### 3.2.2 Business model

For the business model category, support for government carbon-pricing policies (BM9-P&D) and adoption of an internal carbon price for decision-making purposes (BM10-A) are the only areas where consistent activity is observed over the study period. BP and Shell have introduced carbon pricing into decision-making, each setting $40 per ton [88, 94]. Though ExxonMobil’s support for carbon policies dates back to at least 2009 [95], continuous consideration of carbon pricing in internal business decisions (BM10-A) did not occur until 2017.

For other indicators, evidence of a business-model transition toward clean energy is thin, appearing only after 2018. Only the European majors explicitly mention a commitment to shifting beyond fossil fuels to achieve a net-zero-emissions business model (BM1-P&D). In 2019, BP first pledged an ambition to become a net-zero company by 2050 [96]. Outlining a concrete strategy to achieve this through five aims [84], BP is the only major receiving a score for BM2 -A. This strategy includes increasing the proportion of investments in non-fossil fuel businesses and gradually reducing hydrocarbon production (BM3-P&D) and exploration (BM5-P&D). Shell also announced a goal to reach net-zero emissions by 2050 (BM1-P&D). Although it proposed a step-by-step plan to reduce carbon intensity while pledging to refrain from new exploration (BM5-P&D) after 2025, we found no evidence of an explicit plan to achieve such a transition [97].

Several actions are hampering the transition, most notably the refusal to make climate-related commitments or actions to reduce fossil fuel production (BM3-P&D and BM4-A) or fossil fuel exploration (BM5-P&D and BM6-A). While both BP and Shell engaged in new exploration in 2020 [84, 97], this tendency is particularly pronounced at Chevron and ExxonMobil. In 2019, Chevron rejected views that its assets might become stranded, outlining an objective “to be among the most efficient producers” while continuing to develop new fossil fuel reserves [92]. Meanwhile, ExxonMobil emphasized [91]: “The Paris Agreement does not contemplate or require individual companies to decrease production to align with the goal of maintaining global temperature rise to below 2°C. It thereby frames the required energy transition as being related to “society’s demand for energy–not its *supply*” (emphasis added).

For labor force reallocation (BM7-P&D and BM8-A), virtually no activity was observed except BP’s pledge in 2020 of “enabling a just transition for the workforce” [84].

#### 3.2.3 Emissions reduction

The presence of pledges to reach net-zero emissions by 2050 (ER1-P&D) again shows the American majors trailing their European counterparts. While Chevron and ExxonMobil are yet to announce net-zero emission goals, BP and Shell did so in 2019 and 2020 respectively [8, 83, 96, 98]. Furthermore, in 2021 the European majors announced multiple rising targets to achieve net-zero emissions (ER2-A). Shell pledged to reduce the carbon intensity (compared to 2016 levels) of all energy products by 20% by 2030, 45% by 2035, and 100% by 2050 [99].

For scope 3 emissions (ER3-P&D), in 2017 Shell became the first major pledging to reduce the GHG emissions of energy products sold [100, 101]. BP followed from 2019 [8]. No evidence was found however of concrete actions to achieve these pledges (ER4-A). Activity from the two American majors is absent in this field.

All majors have announced pledges to reduce methane emissions (ER5-P&D), but only since 2017 and 2018. All plan to improve energy efficiency and to mitigate flaring by investing in upstream and downstream technologies and by cooperating with industry frameworks such as OGCI. Pledges differ in the details. ExxonMobil pledged in 2017 to reduce absolute levels by 15% by 2020, and to lower flaring by 25% [102], a goal since achieved [103]. However, the other three majors’ intentions only concern intensity [92, 98, 104]. Again, no major has proposed an integrated and concrete roadmap to achieve these emissions reductions (ER6-A). Interestingly, despite growing expectations across the industry to completely eliminate all methane emissions from production [48], no such pledges were observed.

All four majors have lagged in fully disclosing GHG emissions from fossil fuel products produced and sold (ER7-P&D). While each has released data for scope 1 and 2 emissions, disclosure of scope 3 did not begin until 2017 with Chevron. BP followed in 2019, and Shell in 2020. ExxonMobil, however, has refuted the need to disclose scope 3 emissions, stating: “Furthermore, Scope 3 emissions do not provide meaningful insight into the Company’s emission-reduction performance and could be misleading in some respects. For example, increased natural gas sales by ExxonMobil that reduce the amount of coal burned for power generation would result in an overall reduction of global emissions but would increase Scope 3 emissions reported by the Company” [103].

#### 3.2.4 Clean energy investment

Again, the two European majors are more active in clean energy businesses than their American counterparts. But their actions are sporadic and inconsistent.

Regarding the disclosure of annual CAPEX spending for clean energy (CE1-A), Shell and BP have released figures for some years, but not all. BP’s annual data covers only seven years in the study period, while Shell’s cover only three. BP claimed to have invested $1.6 billion in alternative energy in 2011 [105], the highest amount reported. Although later dropping to $750 million in 2020 [84], BP has since pledged to increase annual “low carbon” spending, aiming for $3–4 billion by 2025 and $5 billion by 2030 [84]. Neither ExxonMobil nor Chevron has disclosed information for any year about actual volumes spent, despite claims of increasing investments for low-carbon energy and technologies [85, 91].

Pledges to direct at least 1% of total CAPEX toward clean energy (CE2-P&D) were observed only for the European majors, and these appear only after 2017. Shell pledged to invest $1–2 billion annually into renewables from 2018 to 2020 [106], satisfying the 1% threshold. This increased in 2020 to $2–3 billion annually [97].

No major releases annual investment amounts for clean energy in a consistently transparent format that enables year-to-year tracking. It was, therefore, difficult to verify whether any met the highly conservative 1% threshold (CE3-A). Notwithstanding, BP leads in low-carbon investment, with reported spending on clean energy exceeding 1% of total CAPEX for eight years. Shell also appears to have spent more than 1% for the years it released figures. However historical pledges have been missed. For example, Shell pledged to spend $1–2 billion annually from 2018 to 2020. Yet investments were only disclosed for 2020, with actual spending less than half of pledges ($0.9 billion) [97]. ExxonMobil and Chevron have not disclosed any clean-energy spending volumes during the study period.

#### 3.3.5 Summary of business strategies

To summarize the speed and trajectory of each major’s transition, Fig 3 shows total yearly scores for all indicators, splitting these into pledges and actions. Results show a visible and continuing increase in pledges and disclosure over the study period. This trend is especially pronounced for the European majors after 2016. However, for all majors and in most years, the volume of concrete actions to achieve these is considerably less than pledges. This is notably the case for BP, whose scores from pledges exceed the other three majors by far. For both pledges and actions, the American majors trail their European counterparts significantly. Their laggard status is largely visible with regard to actions, particularly for ExxonMobil.

The European majors’ superior performance is due to greater disclosure of clean-energy investments (CE1-P&D), more consistency in climate cognition (CC1-P&D), and earlier adoption of internal carbon pricing (BM10-A). BP scores highest due to its energy-transition strategies (BM1-P&D and BM2-A) and pledges to reduce hydrocarbon production (BM3-P&D) and exploration (BM5-P&D).

For the American majors, multiple areas have hampered transition, resulting in negative scores. The actions of Chevron and ExxonMobil have remained at or below zero for the entire study period. Regressive strategies include refusing to curb fossil fuel production (BM3-P&D and BM4-A) and exploration (BM5-P&D and BM6-A), the absence of any strategy for net-zero or scope 3 emissions (ER1-P&D to ER4-A), and no investment in clean energy (CE1-A to CE3-A). The American majors thus significantly trail their European counterparts, this gap widening markedly after 2018.

Although Fig 3(A) and 3(B) give the impression of increasing progress towards an energy transition, the relatively lower scores for actions have resulted from several critical areas that contradict pledges. This is even the case for the European pair. For example, BP, which generated the most scores in this analysis, pledged in its 2019 annual report to reduce fossil fuel investment by increasing its non-oil and gas businesses. However it increased its acreage for new exploration access by 58,000 km^2^ in that same year [8]. Further contradicting this intention, several new operating fossil fuel extraction projects started in 2020 [84]. Similarly, Shell also stated an intention to decrease fossil fuel exploration after 2025 [99]. But this target seems to have incited the company to accelerate its exploration program before the “deadline”. Indeed, the undeveloped acreage in Shell’s exploration portfolio increased by around over 38,000 km^2^ in 2020 [97].

### 3.3 Investments: Production, earnings and expenditures

The preceding sections revealed increasing discourse and strategies (mainly pledges) related to climate change and clean energy. The following sections examine investment behavior to verify if a shift away from fossil fuels toward clean energy is actually occurring.

#### 3.3.1 Upstream CAPEX

**Fig 4:**
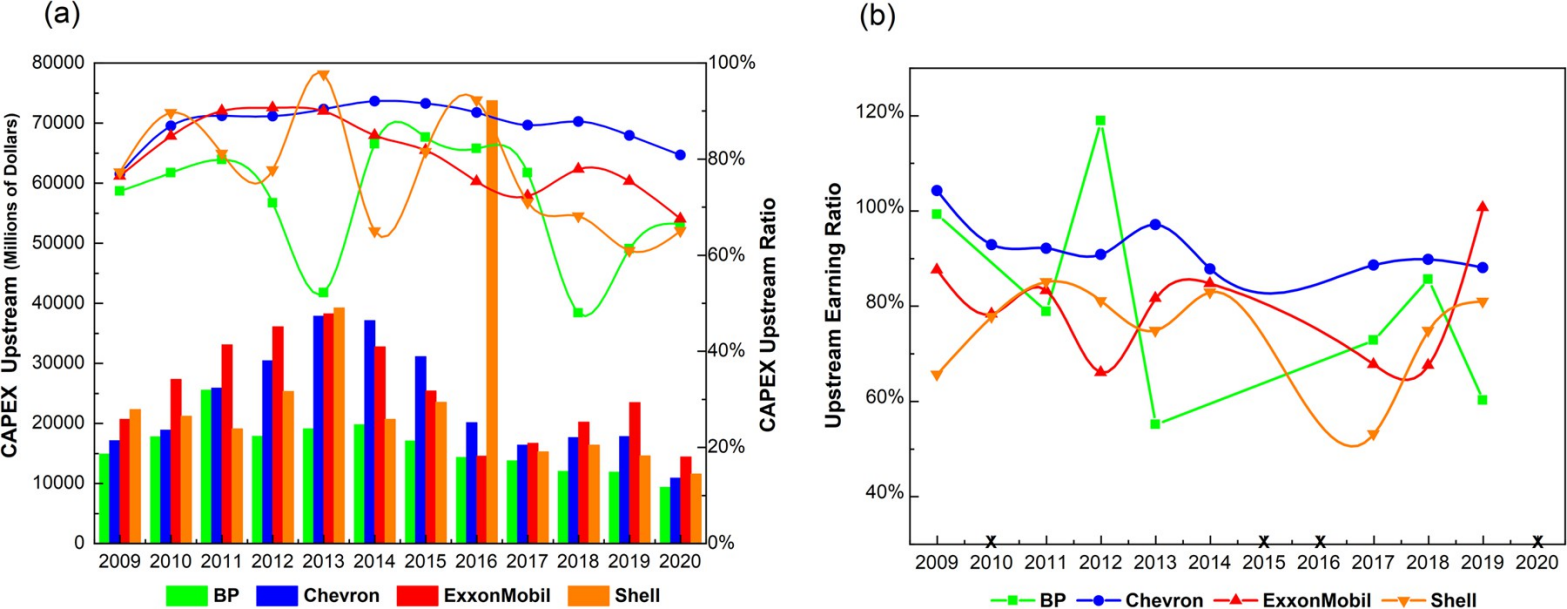
Upstream CAPEX and earnings. (a) Upstream CAPEX (2009~2020) (b) Upstream earnings ratio (2009~2020). Note: BP, Shell, and Chevron incurred negative earnings in 2015 and 2016, due to decreased oil prices. In 2020, all four majors suffered huge losses upstream and across the whole business chain due to reduced oil demand during the COVID-19 pandemic. Trends in these three years should thus not be taken into account. BP also incurred huge losses in 2010 due to the oil leak in the Gulf of Mexico. In 2013, because of the loss of downstream and other business, BP’s total earnings reached only $4,950 million, though upstream earnings were $8,848 million. BP’s earnings ratio during these two years should therefore also be ignored.

Fig 4(A) shows annual upstream CAPEX. Absolute amounts appear as bars, while lines show the portion relative to total CAPEX. In absolute terms, results show a peak of upstream CAPEX spending around 2013, as oil majors battled with price volatility from 2014. This period stimulated a focus on increasing the efficiency of expenditures, instead of increasing overall amounts. In relative terms, and limiting results to 2018–2020, the European majors are notably spending less on upstream capital than their American counterparts. Over the whole period, however, it is difficult to discern a clear trend in CAPEX ratios for the European majors.

For the American majors, relative CAPEX remains constant over the study period (making up 70%–90% of total expenditures over 2016–2020). This is especially so for Chevron, where the CAPEX ratio declined only from 90% in 2016 to 85% in 2019, despite decreasing some $3,000 million in absolute terms (from $20,116m to $17,824m). ExxonMobil has visibly reduced both absolute and relative CAPEX spending since 2013. A concrete explanation for this is not given in annual reports, but statements in 2015 suggest a strategy of focusing on increasing productivity and efficiency to reduce extraction costs [107].

For the European majors, BP’s upstream CAPEX accounts for the smallest proportion. Yet despite declining between 2013 and 2018 (from around 52% to 50%), this rebounded to 60% in 2019 and to 67% in 2020. This is explainable by the start-up of 24 out of 35 new major projects planned since 2016, all on track to deliver 900,000 new barrels of oil equivalent per day by the end of 2021 [8]. For Shell, absolute spending has decreased markedly from 2013 to 2020, from $39,217m to $11,597m. Viewed in relative terms, spending ratios are decreasing, from a peak of nearly 98% in 2013 to 65% in 2020—though punctuated by a rise between 2014 and 2016 due to the acquisition of BG Group, a British multinational oil and gas company [108].

In sum, fluctuations notwithstanding, relative spending trends indicate that upstream exploration and production of oil and gas remain the pillar business for all majors, especially the American majors.

#### 3.3.2 Upstream earnings

Fig 4(B) shows the ratio of upstream earnings from exploration, field development, and production of oil and gas relative to each major’s integrated earnings, including affiliated companies. Annual fluctuations are explained mainly by changing oil and gas prices.

Asides from BP, for the other majors, we find no clear trend to indicate decreasing business-model reliance on upstream fossil fuel production over the study period. The earnings ratios of oil and gas range from 70% to 85%, while Chevron’s range slightly higher, to 90%. Indeed, viewed from 2017 onwards, Chevron, ExxonMobil, and Shell all show a growing share of earnings from upstream fossil fuel businesses. Therefore, as pointed out elsewhere [73], there is no financial rationale to decrease dependence on upstream fossil fuels [109].

BP’s trajectory is exceptional. Ignoring the oil leaks of 2010 and the particular circumstances of 2014 (see the note in Fig 4), BP did not see an increased share of upstream earnings in 2018 and 2019. If we combine the incremental upstream CAPEX ratio shown in Fig 4(A) (the ratio increases from 48% to 61%), we see that the corresponding proportion of generated earnings has fallen (from 86% to 60%). Therefore, BP has a financial rationale to reduce dependence on fossil fuels.

In sum, ignoring BP’s isolated and temporary losses, earnings data indicate that fossil fuels from upstream businesses are still the primary cash engine underpinning each major’s business model.

#### 3.3.3 Upstream production

**Fig 5:**
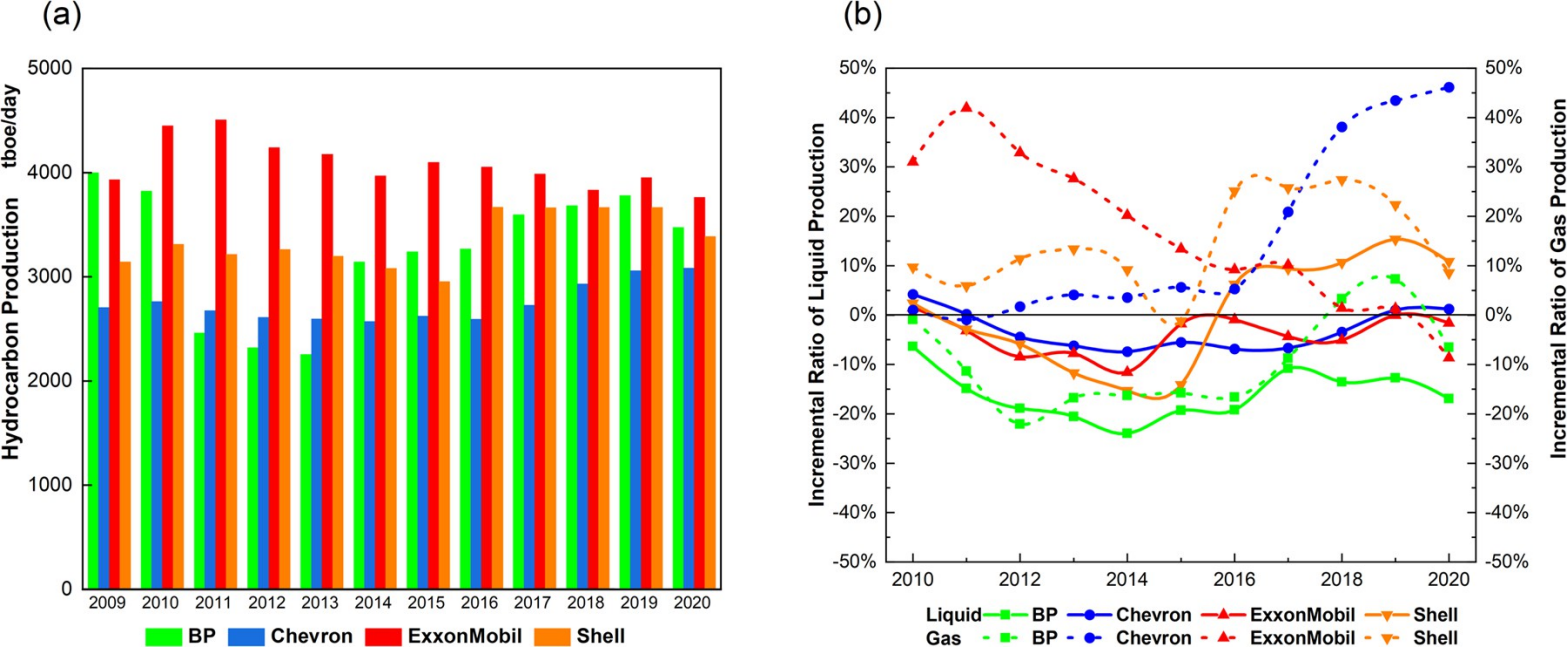
Average daily fossil fuel production. (a) Total hydrocarbon production for gas and oil combined (2009~2020). (b) Incremental production volumes for oil and gas relative to 2009 (2010~2020).

Fig 5(A) shows the average daily volume of hydrocarbon production for liquid and natural gas combined, as units of thousand barrel of oil equivalent (tboe) per day. Fig 5(B) shows the incremental average daily volume of liquid (i.e. oil) and gas production relative to the 2009 baseline. Liquid production appears on the left y-axis as a solid line, gas on the right as a dotted line.

Fig 5(A) indicates that no major has consistently decreased total hydrocarbon production over the study period. If anything, the reverse is true. Shell, BP, and Chevron have increased production volumes, reducing the historical lead of ExxonMobil, the top producer during the study period. ExxonMobil is the only major with a consistently declining trend, dropping from a peak of 4,506 tboe/day in 2011 to 3,761 in 2020, below even 2009 levels.

Now we look at incremental production volumes. Fig 5(B) indicates that after declining temporarily in 2015–2017, each major’s oil and gas production volumes have rebounded to or above baselines; the exception is gas for ExxonMobil. Decreased oil production is explained by depreciated oil prices rather than transition strategies. For instance, in 2016, the West Texas Intermediate oil price dropped to $43.34/barrel against $93.28/barrel in 2014. The COVID-19 pandemic has also created crashes in oil prices since 2020. Despite these disturbances, production by the American majors has been relatively stable compared to the European majors. Indeed, ExxonMobil and Chevron recovered to the 2009 baseline—even in 2020. Shell also shows a massive increase of about 25% from 2015 to 2020.

BP, Shell, and Chevron are increasing production of natural gas. For BP and Chevron, this surged in 2016–2019, indicating increasing faith in this fuel’s prospects in a decarbonizing market [8, 86, 104]. Chevron shows the largest incremental rise, around 40%, from 2016 to 2020. Indeed, we find that all majors have communicated consistently over the study period ambitions to increase gas production for climate mitigation purposes (see each major’s supporting information (CC2)).

Thus, aside from the market-affected declining trend of ExxonMobil, absolute and relative production volumes for the other three majors over the study period fail to show a continuing decrease in hydrocarbon production.

#### 3.3.4 Fossil fuel reserves

**Fig 6:**
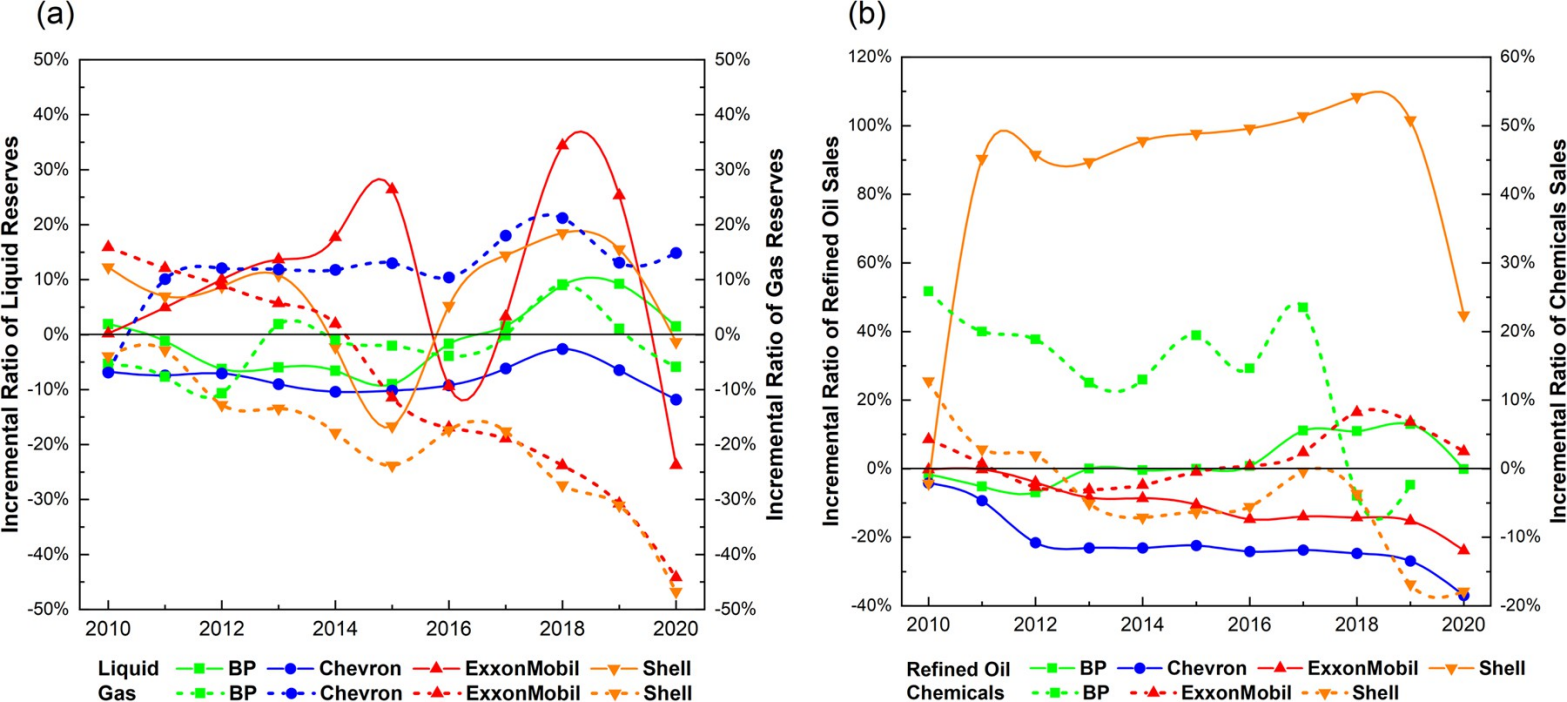
Fossil fuel reserves and petroleum sales. (a) Fossil fuel reserves in the incremental ratio (2010~2020). (b) Petroleum sales in the incremental ratio (2010~2020). Note: Results for Chevron are not included in (b), since its data on chemical sales are not publicly disclosed. BP’s data on chemical sales in 2020 is not released.

Fig 6(A) shows the incremental ratio of annually updated estimates of proved liquid and gas reserves, with 2009 as the baseline. Liquids are shown on the left y-axis as a solid line, gas on the right as a dotted line.

If viewing liquids over the entire study period, despite sharp annual fluctuations, no major shows a persistent downward trend for reserve estimates. Indeed, a declining trend becomes visible only from 2018 onwards. After 2018, all majors show a notable dip, particularly ExxonMobil. However, BP and Shell showed a clear growth pattern from 2015 to 2019, while ExxonMobil’s reserve estimates surge between 2016 and 2018.

If viewing gas reserves over the entire study period, a consistent plummeting trend is visible for ExxonMobil and Shell. Although its precise reasons are not disclosed, Shell mentioned in its 2020 annual report that the high cost of fossil fuel production has negatively impacted entitlement to proved reserves [97]. In contrast, BP and Chevron show a rising trend. The tendency toward higher gas-reserve estimates is strongest for Chevron. This major has opted to expand the volume of gas reserves in its portfolio for future production, with these making up more than half of total reserves in 2019 [110].

Given the continuing possession of hydrocarbon reserves under holding, we assume that each major plans to develop these gradually in coming years to avoid these assets becoming stranded in the transition to a carbon-free future.

#### 3.3.5 Downstream petroleum sales

Fig 6(B) indicates the incremental downstream petroleum sales ratio, including refined oil and chemicals, with 2009 as the baseline. Refined oil appears on the left y-axis as a solid line, with chemicals on the right as a dotted line.

Shell and BP show a tendency toward increasing oil sales until 2019 and the global pandemic. Shell has grown refined-oil sales substantially during the study period, rising more than 100% until 2018. Indeed, as recently as 2017 and 2018, Shell explicitly stated it considers downstream oil products as an important “cash engine” [100, 101]. BP also shows a consistent but gradual trend toward rising refined-oil sales until 2019.

ExxonMobil and Chevron show a contrasting pattern, trending toward decreasing oil sales. For ExxonMobil, this trend is accompanied by a distinct increase in downstream chemical sales after 2013. This appears to reflect a previously stated view [111] that long-term prospects for high margins in refined oil would remain weak, while global demand for chemicals would grow significantly, driven principally by improving prosperity in developing countries.

#### 3.3.6 Renewables investment

We use third-party data to show trends in renewables and clean energy investment, since no major periodically discloses these amounts.

**Fig 7:**
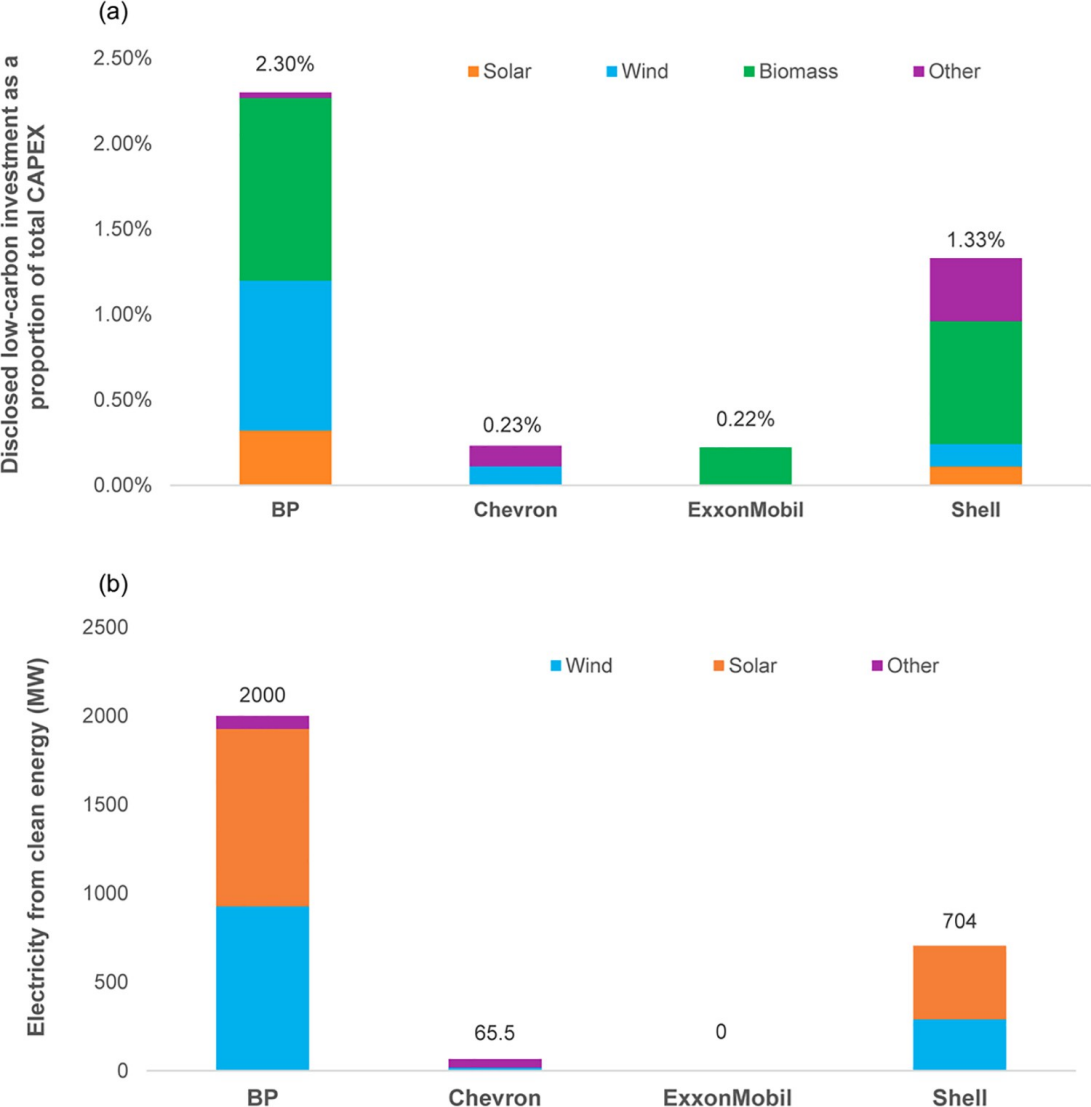
Low-carbon energy investment and electricity generation from clean energy. (a) Disclosed investment in low-carbon energy production and development, as a proportion of total CAPEX (2010–Q3 2018). Source: CDP Investor Research [81]. Note: “Other” indicates hydro; carbon capture, utilization, and storage (CCUS); frontier power; and smart technologies. (b) Electricity generation from clean energy (2009–2019). Source: S&P Global Platts [82]. Note: “Other” indicates other renewables used to generate electricity.

Fig 7(A) provides a rough picture of cumulative clean energy investment (reflecting spending on assets and venture capital) as a proportion of total CAPEX. The European majors lead investment trends. BP has spent more than 2% of CAPEX on clean energy, mostly biomass and wind, over the study period. Shell has spent 1.33%, mostly on biomass. The American majors lag well behind: total CAPEX spending makes up only 0.22% and 0.23%, far below the industry average of around 0.5%–0.8% between 2015 and 2019, reported by IEA [4].

Interpreting these results requires caution. Not only is it unclear if these amounts depict R&D, business development or actual energy production, moreover, carbon capture and storage technologies (shown as “other”) makes up a large part of low carbon investment for Chevron and Shell.

Fig 7(B) shows the cumulative size of clean electricity generation capacity. Again, the European majors, notably BP, lead the group of four. The American majors trail far behind. Glaringly, ExxonMobil generated no clean energy during the decade, instead stating an unwillingness to invest in solar and wind [91]. But even BP’s global renewables capacity, the largest of the four majors, amounts to only 2,000 MW—the equivalent of around two large gas-fired power plants.

Piecing together CAPEX and electricity generation amounts, we find no evidence to suggest any major has entered the renewables market at a scale that would indicate a shift away from fossil fuels.

## 4. Conclusions and discussion

This study collected twelve-years of quantitative and qualitative data to examine if BP, Chevron, ExxonMobil and Shell are decarbonizing and shifting from fossil fuels to clean energy. We comprehensively and objectively evaluated this from three perspectives: (i) discourse, (ii) business strategies, and (iii) investments.

The discourse analysis revealed a distinct increase in keywords in annual reports related to climate change and clean energy, particularly by the European majors, BP and Shell. Similarly, the business strategy analysis also revealed contrasting behavior between the American majors, Chevron and ExxonMobil, and their European counterparts. Over the study period, the European majors have more consistently acknowledged climate science, participated earlier in industry climate-change frameworks, adopted internal carbon pricing, spent and pledged more on clean energy, and recently set net-zero transition and energy product decarbonization goals. Trailing far behind, the American majors continuously exhibit defensive attitudes to renewables investment and the need to shift from fossil fuels, explicitly stating ambitions to grow rather than reduce hydrocarbon production.

For all majors, however, we caution that most strategy scores have come from “low-hanging fruit” in the form of pledges and disclosure. These include simple statements of support for climate science or carbon pricing and disclosure of GHG emissions data. Thus, shifting core businesses away from fossil fuels to clean energy still requires that each major formulates concrete strategies to translate pledges into actions.

Moreover, we found that some actions contradict pledges. This especially concerns intentions to curb the production of fossil fuels as well as reduce exploration and new developments. This worrying trend of acting contrary to pledges and public statements has also been highlighted by other sources. This includes reports that all four majors continue to lobby governments to hamper or weaken carbon pricing policies [112–114], to secure favorable fiscal support, and to weaken environmental regulations [3, 29, 115, 116]. Also in the goal of obstructing the progress of decarbonization, they continue to redirect the responsibility for reducing GHG emissions to consumers [25, 117] while diffusing misleading advertisements that fossil fuels (especially gas) are green [30, 118, 119] and exaggerating the scale of clean energy investments [119].

The analysis of financial behavior generated a picture even more sharply misaligned with tendencies toward increased green discourse. This failed to show any major comprehensively transitioning its core business model away from fossil fuels. Bar year-to-year fluctuations and influence of the pandemic, we did not observe a clear trend toward lower fossil fuel production, less business-model reliance on upstream earnings, and declining fossil fuel reserves. Indeed, the European majors increased combined oil and gas production and reserve estimates for liquids over 2015–19. Similarly, gas reserve estimates for BP and Chevron rose. We also note continuing ambitions stated by all majors to increase gas production. Finally, the analysis of investments in renewables revealed no evidence to suggest any major has entered the renewables market at a scale that would indicate a shift away from fossil fuels.

Given the mismatch between discourse, pledges, actions and investments, aligning with recent studies [31, 63], we conclude that no major is currently on the way to a clean energy transition. Thus, when weighing the authenticity of claims to be decarbonizing or moving away from fossil fuels, stakeholders and policymakers should consider the past actions and investments we have examined. Mitigating further dangerous warming requires these majors to urgently transform their fossil-fuel-based business models rather than merely increase discourse and pledges. Furthermore, for clean-energy investments, there is a need for more transparency on precise annual spending and on each major’s definition of “renewables”, “low-carbon”, and “clean energy”. Until the three areas of discourse, actions and investment behavior are brought into alignment, we conclude that accusations of greenwashing by oil majors are well-founded [34, 113].

Clarifying the factors that have influenced the contrasting behavior of the American and European majors was beyond this study’s scope. However, based on insights provided by previous literature, we speculate that trends found in this study reflect the historically more aggressive emission reduction targets and climate policies of governments in European countries [3, 31, 50, 120–122]. Conversely, the regulatory climate in the home country of the American majors was significantly weakened during the Trump administration over 2016 to 2020 [3]. BP and Shell have also been observed to have historically possessed more pro- leadership and management structures that more readily engage in climate issues [121, 122]. Furthermore, more experience in renewables might also give them more confidence to profit from future clean energy markets, since their American competitors lack a comparable investing history [121].

Our findings of a mismatch between words, actions and investments prompt a need to understand the factors that incite the majors to talk about the energy transition rather than pursue it. Green discourse and pledges provide the benefit of alleviating pressure from society [38, 119, 120]. Not only can this generate a positive image of the company for consumers [50, 123], such messages can prolong the social license to operate, providing valuable time for the majors to continue their core fossil fuel business [119, 119, 124]. Financial factors are also important. Large investments in renewables are generally less profitable for the majors than traditional core businesses, and such activities place them in competition with specialized players [4, 82, 125]. Moreover, any shift away from traditional businesses that are currently profitable will initiate an irreversible process of writing down the value of existing fossil fuels assets and reserves, carrying significant consequences for share prices [3]. The rebound of the oil and gas market after the COVID-19 pandemic in 2021 also offers the majors more confidence in future benefits [59, 126–128]. Slowing down the transition to clean energy is hence profitable for the boards of these majors [3, 129]. To counter these complex and interwoven forces, policymakers must reform market conditions and abolish subsidies that continue to incentivize investments in the extraction and consumption of fossil fuels [55, 130].

Methodological limitations provide important opportunities for future research. Given the absence of first-hand data about annual spending on clean energy in a consistent year-to-year format, researchers might attempt to produce such data and define standards on what investment targets should be considered “clean energy”. Future studies could also refine our indicators of transition activity and develop finer-grained quantitative measurements of progressive and regressive behavior, also allowing integration of qualitative data.

## Supporting information

S1 File Discourse analysis (method and omitted terms). (PDF)

S2 File Indicator scoring criteria. (XLSX)

S1 Dataset Discourse analysis results (all majors). (XLSX)

S2 Dataset Business strategy BP. (XLSX)

S3 Dataset Business strategy Chevron. (XLSX)

S4 Dataset Business strategy ExxonMobil. (XLSX)

S5 Dataset Business strategy Shell. (XLSX)

S6 Dataset Financial analysis (all majors). (XLSX)

## References

[1] Tong D, Zhang Q, Zheng Y, Caldeira K, Shearer C, Hong C, et al. Committed emissions from existing energy infrastructure jeopardize 1.5°C climate target. Nature. 2019; 572:373–7. doi: 10.1038/s41586-019-1364-3 . 31261374 PMC6697221

[2] Heede R. Tracing anthropogenic carbon dioxide and methane emissions to fossil fuel and cement producers, 1854–2010. Climatic Change. 2014; 122:229–41. doi: 10.1007/s10584-013-0986-y

[3] Kenner D, Heede R. White knights, or horsemen of the apocalypse? Prospects for Big Oil to align emissions with a 1.5°C pathway. Energy Research & Social Science. 2021:102049. doi: 10.1016/j.erss.2021.102049

[4] IEA. The Oil and Gas Industry in Energy Transitions. Insight from IEA analysis. IEA 2020 [updated January 2020; cited 25 Oct 2020]. Available from: https://www.iea.org/reports/the-oil-and-gas-industry-in-energy-transitions.

[5] Lenzen M, Li M, Malik A, Pomponi F, Sun Y-Y, Wiedmann T, et al. Global socio-economic losses and environmental gains from the Coronavirus pandemic. PLoS ONE. 2020; 15:e0235654. Epub 2020/07/09. doi: 10.1371/journal.pone.0235654 . 32645023 PMC7347123

[6] Gandel S. Exxon Mobil dropped from the Dow after nearly a century, replaced by Salesforce [updated 25 Aug 2020; cited 11 Mar 2021]. Available from: https://www.cbsnews.com/news/dow-jones-exxon-mobil-pfizer-raytheon-replaced-salesforce-amgen-honeywell/.

[7] BBC. Elon Musk becomes world’s richest person as wealth tops $185bn [updated 8 Jan 2021; cited 8 Aug 2021]. Available from: https://www.bbc.com/news/technology-55578403.

[8] BP. Annual report and form 20f 2019. BP [updated 2020; cited 16 Sep 2020]. Available from: https://www.bp.com/content/dam/bp/business-sites/en/global/corporate/pdfs/investors/bp-annual-report-and-form-20f-2019.pdf.

[9] Meckling J, Nahm J. The politics of technology bans: Industrial policy competition and green goals for the auto industry. Energy Policy. 2019; 126:470–9. doi: 10.1016/j.enpol.2018.11.031

[10] Ayling J, Gunningham N. Non-state governance and climate policy: the fossil fuel divestment movement. Climate Policy. 2017; 17:131–49. doi: 10.1080/14693062.2015.1094729

[11] Green F. Anti-fossil fuel norms. Climatic Change. 2018; 150:103–16. doi: 10.1007/s10584-017-2134-6

[12] Blondeel M, van de Graaf T. Toward a global coal mining moratorium? A comparative analysis of coal mining policies in the USA, China, India and Australia. Climatic Change. 2018; 150:89–101. doi: 10.1007/s10584-017-2135-5

[13] Frumhoff PC, Heede R, Oreskes N. The climate responsibilities of industrial carbon producers. Climatic Change. 2015; 132:157–71. doi: 10.1007/s10584-015-1472-5

[14] Volcovici V. Minnesota sues Exxon, Koch and API for being ’deceptive’ on climate change. 2020 Jun 25 [updated 2020 Jun 25; cited 2020 Jun 26].

[15] Congress of United States. Analysis of the Fossil Fuel Industrys Legislative Lobbying and Capital Expenditures Related to Climate Change—Staff Memo (10.28.21). 2021 [cited 28 Nov 2021]. Available from: https://oversight.house.gov/sites/democrats.oversight.house.gov/files/Analysis%20of%20the%20Fossil%20Fuel%20Industrys%20Legislative%20Lobbying%20and%20Capital%20Expenditures%20Related%20to%20Climate%20Change%20-%20Staff%20Memo%20%2810.28.21%29.pdf.

[16] Maloney C. Oversight Committee Launches Investigation of Fossil Fuel Industry Disinformation on Climate Crisis. 2021 [updated 17 Sep 2021; cited 28 Nov 2021]. Available from: https://oversight.house.gov/news/press-releases/oversight-committee-launches-investigation-of-fossil-fuel-industry.

[17] Walsh D, Pierre J. Oil companies face ’big tobacco moment’ in Congress over their climate policies. NPR. 2021 Oct 28. Available from: https://www.npr.org/2021/10/28/1049287610/oil-companies-face-big-tobacco-moment-in-congress-over-their-climate-policies [updated 2021 Oct 28; cited 2021 Nov 28].

[18] IEA. Energy Technology Perspectives 2020 –Analysis—IEA [updated 26 Apr 2021; cited 26 Apr 2021]. Available from: https://www.iea.org/reports/energy-technology-perspectives-2020.

[19] Fattouh B, Poudineh R, West R. The rise of renewables and energy transition: what adaptation strategy exists for oil companies and oil-exporting countries. Energy Transit. 2019; 3:45–58. doi: 10.1007/s41825-019-00013-x

[20] Breetz H, Mildenberger M, Stokes L. The political logics of clean energy transitions. Business and Politics. 2018; 20:492–522. doi: 10.1017/bap.2018.14

[21] Coffin M. Absolute Impact: Why oil majors’ climate ambitions fall short of Paris limits—Carbon Tracker Initiative. 2020 [updated 8 Aug 2021; cited 20217/8].

[22] Mulvey K, Piepenburg J, Goldman G, Frumhoff PC. The Climate Accountability Scorecard. Ranking Major Fossil Fuel Companies on Climate Deception, Disclosure, and Action. Union of Concerned Scientists 2017 [updated 2017; cited 3 Feb 2019]. Available from: https://www.ucsusa.org/resources/climate-accountability-scorecard.

[23] Pinko N, Mulvey K, Ekwurzel B, Frumhoff PC, Hurd N, Sideris J. The 2018 Climate Accountability Scorecard. Insufficient Progress from Major Fossil Fuel Companies. Union of Concerned Scientists 2019 [updated 2019; cited 20 Nov 2019]. Available from: www.ucsusa.org/climatescorecard.

[24] Supran G, Oreskes N. Assessing ExxonMobil’s climate change communications (1977–2014). Environ Res Lett. 2017; 12:84019. doi: 10.1088/1748-9326/aa815f

[25] Supran G, Oreskes N. Rhetoric and frame analysis of ExxonMobil’s climate change communications. One Earth. 2021; 4:696–719. doi: 10.1016/j.oneear.2021.04.014

[26] Grasso M. Oily politics: A critical assessment of the oil and gas industry’s contribution to climate change. Energy Research & Social Science. 2019; 50:106–15. doi: 10.1016/j.erss.2018.11.017

[27] Jaworska S. Change But no Climate Change: Discourses of Climate Change in Corporate Social Responsibility Reporting in the Oil Industry. International Journal of Business Communication. 2018; 55:194–219. doi: 10.1177/2329488417753951

[28] Solnit R. Big oil coined ‘carbon footprints’ to blame us for their greed. Keep them on the hook | Rebecca Solnit. The Guardian. 2021 Aug 23. Available from: https://www.theguardian.com/commentisfree/2021/aug/23/big-oil-coined-carbon-footprints-to-blame-us-for-their-greed-keep-them-on-the-hook [updated 2021 Aug 23; cited 2021 Nov 28].

[29] Map Influence. Big Oil’s Real Agenda on Climate Change: How the oil majors have spent $1bn since Paris on narrative capture and lobbying on climate [updated 2019]. Available from: https://influencemap.org/report/How-Big-Oil-Continues-to-Oppose-the-Paris-Agreement-38212275958aa21196dae3b76220bddc.

[30] Map Influence. Climate Change and Digital Advertising. The Oil & Gas Industry’s Digital Advertising Strategy. 2021 [updated 8 Aug 2021; cited 8 Aug 2021]. Available from: https://influencemap.org/report/Climate-Change-and-Digital-Advertising-a40c8116160668aa2d865da2f5abe91b#.

[31] Green JF, Hadden J, Hale T, Mahdavi P. Transition, Hedge, or Resist? Understanding Political and Economic Behavior toward Decarbonization in the Oil and Gas Industry. Review of International Political Economy. 2021. doi: 10.1080/09692290.2021.1946708

[32] Scanlan SJ. Framing fracking: scale-shifting and greenwashing risk in the oil and gas industry. Local Environment. 2017; 22:1311–37. doi: 10.1080/13549839.2017.1345877

[33] Sheehan Kim. This Ain’t Your Daddy’s Greenwashing: An Assessment of the American Petroleum Institute’s Power Past Impossible Campaign: The Paris Agreement and Climate Justice. Intellectual Property and Clean Energy.; 2018. pp. 301–21.

[34] Brulle RJ, Aronczyk M, Carmichael J. Corporate promotion and climate change: an analysis of key variables affecting advertising spending by major oil corporations, 1986–2015. Climatic Change. 2020; 159:87–101. doi: 10.1007/s10584-019-02582-8

[35] Gatti L, Pizzetti M, Seele P. Green lies and their effect on intention to invest. Journal of Business Research. 2021; 127:228–40. doi: 10.1016/j.jbusres.2021.01.028

[36] Jong MDT de, Huluba G, Beldad AD. Different Shades of Greenwashing: Consumers’ Reactions to Environmental Lies, Half-Lies, and Organizations Taking Credit for Following Legal Obligations. Journal of Business and Technical Communication. 2020; 34:38–76. doi: 10.1177/1050651919874105

[37] Map Influence. Big Tech and Climate Policy. Are the Technology Giants Deploying Political Capital on Climate Change. Influence Map [updated 28 Jan 2021; cited 28 Jan 2021]. Available from: https://influencemap.org/EN/report/Big-Tech-and-Climate-Policy-afb476c56f217ea0ab351d79096df04a.

[38] Shojaeddini E, Naimoli S, Ladislaw S, Bazilian M. Oil and gas company strategies regarding the energy transition. Prog Energy. 2019; 1:12001. doi: 10.1088/2516-1083/ab2503

[39] Naimoli S, Ladislaw S. Oil and Gas Industry Engagement on Climate Change. Drivers, Actions, and Path Forward. CSIS Energy and National Security Program; 2019.

[40] Pickl MJ. The renewable energy strategies of oil majors–From oil to energy. Energy Strategy Reviews. 2019; 26:100370. doi: 10.1016/j.esr.2019.100370

[41] Tong D, Trout K, Mckinnon H, Stockman L. Big Oil Reality Check. Assessing Oil And Gas Company Climate Plans. Oil Change International [updated 2020; cited 2 Oct 2020]. Available from: http://priceofoil.org/content/uploads/2020/09/OCI-Big-Oil-Reality-Check-vF.pdf.

[42] Shaw F, Donovan C. Assessing the preparedness of major oil and gas companies for a low-carbon energy transition. Journal of Insurance and Financial Management. 2021:Vol. 4, Issue 3 (2021) 16–48. Available from: https://journal-of-insurance-and-financial-management.com/index.php/JIFM/article/view/181/pdf.

[43] Bach M. The oil and gas sector: from climate laggard to climate leader. Environmental Politics. 2019; 28:87–103. doi: 10.1080/09644016.2019.1521911

[44] Carley S, Konisky DM. The justice and equity implications of the clean energy transition. Nat Energy. 2020; 5:569–77. doi: 10.1038/s41560-020-0641-6

[45] Airò Farulla G, Tumminia G, Sergi F, Aloisio D, Cellura M, Antonucci V, et al. A Review of Key Performance Indicators for Building Flexibility Quantification to Support the Clean Energy Transition. Energies. 2021; 14:5676. doi: 10.3390/en14185676

[46] IEA. Net Zero by 2050. A Roadmap for the Global Energy Sector. 2021 [updated 29 Jul 2021; cited 29 Jul 2021]. Available from: https://www.iea.org/reports/net-zero-by-2050.

[47] Rajaraman A, Ullman JD. Data Mining. In: Rajaraman A, Ullman JD, editors. Mining of Massive Datasets. Cambridge: Cambridge University Press; 2011. pp. 1–17.

[48] OGCI. OGCI Progress Report 2020. 2021 [cited 2 Mar 2021]. Available from: https://oilandgasclimateinitiative.com/wp-content/uploads/2020/12/OGCI-Progress-Report-2020.pdf.

[49] Carbon Tracker Initiative. Breaking the Habit—Why none of the large oil companies are “Paris-aligned”, and what they need to do to get there—Carbon Tracker Initiative. 2019 [updated 10 Mar 2021; cited 1 Mar 2020]. Available from: https://carbontracker.org/reports/breaking-the-habit/.

[50] Skjaerseth JB, Skodvin T. Climate change and the oil industry. Common problem different strategies. Manchester University Press; 2003.

[51] Johnsson F, Karlsson I, Rootzén J, Ahlbäck A, Gustavsson M. The framing of a sustainable development goals assessment in decarbonizing the construction industry–Avoiding “Greenwashing”. Renewable and Sustainable Energy Reviews. 2020; 131:110029. doi: 10.1016/j.rser.2020.110029 34234615 PMC7352113

[52] Grasso M. Towards a broader climate ethics: Confronting the oil industry with morally relevant facts. Energy Research & Social Science. 2020; 62:101383. doi: 10.1016/j.erss.2019.101383

[53] Johnston RJ, Blakemore R, Bell R. The Role Of Oil And Gas Companies In The Energy he Role Of Oil And Gas Companies In The Energy Transition. Atlantic Council [updated 2020; cited 10 Sep 2020]. Available from: https://www.atlanticcouncil.org/wp-content/uploads/2020/01/OGT-final-web-version.pdf.

[54] Fouquet R. Historical energy transitions: Speed, prices and system transformation. Energy Research & Social Science. 2016; 22:7–12. doi: 10.1016/j.erss.2016.08.014

[55] Nicolas G, Le Billon P. Climate change and fossil fuel production cuts: assessing global supply-side constraints and policy implications. Climate Policy. 2020; 20:888–901. doi: 10.1080/14693062.2020.1725409

[56] Zhang MM, Wang Q, Zhou D, Ding H. Evaluating uncertain investment decisions in low-carbon transition toward renewable energy. Applied Energy. 2019; 240:1049–60. doi: 10.1016/j.apenergy.2019.01.205

[57] Erickson P, Lazarus M, Piggot G. Limiting fossil fuel production as the next big step in climate policy. Nat Clim Chang. 2018; 8:1037–43. doi: 10.1038/s41558-018-0337-0

[58] Carbon Tracker Initiative. Balancing the Budget: Why deflating the carbon bubble requires oil & gas companies to shrink—Carbon Tracker Initiative. Carbon Tracker Initiative 2020 [updated 10 Mar 2021; cited 20 Mar 2020]. Available from: https://carbontracker.org/reports/balancing-the-budget/.

[59] Mäkitie T, Normann HE, Thune TM, Sraml Gonzalez J. The green flings: Norwegian oil and gas industry’s engagement in offshore wind power. Energy Policy. 2019; 127:269–79. doi: 10.1016/j.enpol.2018.12.015

[60] IEA. Achieving net-zero emissions by 2050 –World Energy Outlook 2020 –Analysis—IEA. IEA 2020 [updated 2020/10; cited 11 Jan 2021]. Available from: https://www.iea.org/reports/world-energy-outlook-2020/achieving-net-zero-emissions-by-2050.

[61] IPCC. Global warming of 1.5°C. An IPCC Special Report on the impacts of global warming of 1.5°C above pre-industrial levels and related global greenhouse gas emission pathways, in the context of strengthening the global response to the threat of climate change, sustainable development, and efforts to eradicate poverty. IPCC 2018 [cited 13 Aug 2019]. Available from: https://www.ipcc.ch/site/assets/uploads/sites/2/2019/06/SR15_Full_Report_High_Res.pdf.

[62] Hertwich EG, Wood R. The growing importance of scope 3 greenhouse gas emissions from industry. Environ Res Lett. 2018; 13:104013. doi: 10.1088/1748-9326/aae19a

[63] Dietz S, Gardiner D, Jahn V, Noels J. How ambitious are oil and gas companies’ climate goals. Science. 2021; 374:405–8. Epub 2021/10/21. doi: 10.1126/science.abh0687 . 34672760

[64] van der Ploeg F. Macro-financial implications of climate change and the carbon transition. European Central Bank. 2020. Available from: https://www.ecb.europa.eu/pub/conferences/shared/pdf/20201111_ECB_Forum/academic_paper_vanderPloeg.pdf.

[65] World Benchmarking Alliance. WBA Climate and Energy Benchmark. Methodology report Electric utilities sector [updated 2020; cited 18 Jul 2020]. Available from: https://assets.worldbenchmarkingalliance.org/app/uploads/2020/09/WBA-Climate-and-Energy-Benchmark-methodology-EU.pdf.

[66] Piggot G, Verkuijl C, van Asselt H, Lazarus M. Curbing fossil fuel supply to achieve climate goals. Climate Policy. 2020; 20:881–7. doi: 10.1080/14693062.2020.1804315

[67] CDP. CDP Climate Change 2019 Scoring Methodology. 2020 [updated 2020; cited 12 Sep 2020]. Available from: https://guidance.cdp.net/en/guidance?cid = 8&ctype = theme&idtype = ThemeID&incchild = 1&microsite = 0&otype = ScoringMethodology&tags = TAG-599%2CTAG-605%2CTAG-585.

[68] Bashir MF, MA B, Shahbaz M, Jiao Z. The nexus between environmental tax and carbon emissions with the roles of environmental technology and financial development. PLoS ONE. 2020; 15:e0242412. Epub 2020/11/25. doi: 10.1371/journal.pone.0242412 . 33237920 PMC7688139

[69] Lu H, Guo L, Zhang Y. Oil and gas companies’ low-carbon emission transition to integrated energy companies. Sci Total Environ. 2019; 686:1202–9. doi: 10.1016/j.scitotenv.2019.06.014 . 31412516

[70] World Economic Forum. Fostering Effective Energy Transition 2019. World Economic Forum [updated 2019; cited 22 May 2020]. Available from: https://www.weforum.org/reports/fostering-effective-energy-transition-2019.

[71] Fogarassy C, Neubauer É, Mansur H, Tangl A, Oláh J, Popp J. The main transition management issues and the effects of environmental accounting on financial performance-with focus on cement industry. Administratie si Management Public. 2018:52–66.

[72] Kulagin VA, Grushevenko DA, Kapustin NO. Fossil fuels markets in the “energy transition” era. Russian Journal of Economics. 2020; 6:424–36. doi: 10.32609/j.ruje.6.55177

[73] Beck C, Bellone D, Hall S, Kar J, Olufon D. The big choices for oil and gas in navigating the energy transition.; 2021/3/10.

[74] Aminjonov F. Policy Innovations and Rationale for Sustainable Energy Transition in the UAE. Social Science Quarterly. 2020; 101:2398–412. doi: 10.1111/ssqu.12909

[75] Newell P, Simms A. Towards a fossil fuel non-proliferation treaty. Climate Policy. 2020; 20:1043–54. doi: 10.1080/14693062.2019.1636759

[76] Stockman L. Burning the Gas ‘Bridge Fuel’ Myth Why gas is not clean, cheap or necessary [updated 2019; cited 8 Aug 2020]. Available from: http://priceofoil.org/2019/05/30/gas-is-not-a-bridge-fuel/.

[77] van der Ploeg F. Fossil fuel producers under threat. Oxford Centre for the Analysis of Resource Rich Economies. 2016; 32:206–22. doi: 10.1093/oxrep/grw004

[78] Grant A, Coffin M. How diverging oil and gas company strategies link to stranded asset risk [updated 2020]. Available from: https://carbontracker.org/reports/fault-lines/.

[79] Krane J. Climate change and fossil fuel: An examination of risks for the energy industry and producer states. MRS energy sustain. 2017; 4. doi: 10.1557/mre.2017.3

[80] Pinko N. BP’s 2019 Annual Report Holds Clues to How It Will Meet Grand Climate Goals (But No Definitive Answer). Union of Concerned Scientists 2020 [cited 27 Mar 2020]. Available from: https://blog.ucsusa.org/nicole-pinko/bps-2019-annual-report-holds-clues-to-how-it-will-meet-grand-climate-goals-but-no-definitive-answer.

[81] Fletcher L, Crocker T, Smyth J, Marcell K. Beyond the cycle: which oil and gas companies are ready for the low-carbon transition.; 2018.

[82] Edwardes-Evans H, Burgess J, Emma Slawinski. Cross currents: Big oil and the energy transition. S&P Global Platts. 2020 Apr 21. Available from: https://www.spglobal.com/platts/en/market-insights/blogs/electric-power/042120-cross-currents-big-oil-and-the-energy-transition [updated 2020 Apr 21; cited 2021 Jun 5].

[83] Shell. Shell annual report 2019. Shell [updated 2020; cited 2020 August 13]. Available from: https://reports.shell.com/annual-report/2019/.

[84] BP. Annual Report and Form 20f 2020. 2021 [cited 1 May 2021]. Available from: https://www.bp.com/content/dam/bp/business-sites/en/global/corporate/pdfs/investors/bp-annual-report-and-form-20f-2020.pdf.

[85] Chevron. Chevron Annual Report 2020. 2021 [cited 1 May 2021]. Available from: https://www.chevron.com/-/media/chevron/annual-report/2020/documents/2020-Annual-Report.pdf.

[86] Chevron. Chevron 2016 Corporate Responsibility Report Highlights. 2017 [cited 16 May 2019]. Available from: https://www.chevron.com/-/media/shared-media/documents/2016-corporate-responsibility-report.pdf.

[87] Shell. Annual Report 2009. 2010 [cited 13 May 2020]. Available from: https://www.shell.com/about-us/annual-publications/annual-reports-download-centre/_jcr_content/par/tabbedcontent_f645/tab_370040026/textimage.stream/1509452626404/b2a6ec7d2026989f2b2837b1dcd9d179b9f794d7/annual-report-2009.pdf.

[88] BP. BP Sustainability Review 2010. 2011 [cited 21 Jun 2020]. Available from: https://www.bp.com/content/dam/bp/business-sites/en/global/corporate/pdfs/sustainability/archive/archived-reports-and-translations/2010/bp_sustainability_review_2010.pdf.

[89] BP. BP Annual Report and Form 20-F 2011. BP 2012 [cited 3 Jul 2020]. Available from: https://www.bp.com/content/dam/bp/business-sites/en/global/corporate/pdfs/investors/bp-annual-report-and-form-20f-2011.pdf.

[90] ExxonMobil. Energy and Carbon—Managing the Risks. ExxonMobil [updated 2014; cited 6 Sep 2020]. Available from: http://www.lawandenvironment.com/wp-content/uploads/sites/5/2014/04/Report-Energy-and-Carbon-Managing-the-Risks1.pdf.

[91] ExxonMobil. 2021 Energy & Carbon Summary. 2021 [cited 1 May 2021]. Available from: https://corporate.exxonmobil.com/-/media/Global/Files/energy-and-carbon-summary/Energy-and-carbon-summary.pdf.

[92] Chevron. Update to Climate Change Resilience [updated 2019; cited 16 Aug 2020]. Available from: https://www.chevron.com/-/media/shared-media/documents/update-to-climate-change-resilience.pdf.

[93] BP. Sustainability Report. BP [updated 2016; cited 16 Oct 2020]. Available from: https://www.bp.com/content/dam/bp/business-sites/en/global/corporate/pdfs/sustainability/archive/archived-reports-and-translations/2016/bp-sustainability-report-2016.pdf.

[94] Shell. Annual Report 20-F 2010 [cited 5 Aug 2020]. Available from: https://www.shell.com/about-us/annual-publications/annual-reports-download-centre/_jcr_content/par/tabbedcontent_f645/tab/textimage.stream/1509452607868/5cab46fcc603585e21b47aa28069e27393feff2a/annual-report-2010.pdf.

[95] ExxonMobil. ExxonMobil 2009 Citizenship Report [cited 5 Aug 2020]. Available from: https://www.scribd.com/document/56345424/1275354520-ExxonMobil-2009-Citizenship-Report.

[96] BP. Sustainability Report 2019. BP [updated 2020; cited 16 Oct 2020]. Available from: https://www.bp.com/content/dam/bp/business-sites/en/global/corporate/pdfs/sustainability/group-reports/bp-sustainability-report-2019.pdf.

[97] Shell. Shell annual report 2020. 2021 [cited 1 May 2021]. Available from: https://reports.shell.com/annual-report/2020/servicepages/downloads/files/shell-annual-report-2020.pdf.

[98] Shell. Sustainability Report 2019. Royal Dutch Shell plc [updated 2020; cited 16 Oct 2020]. Available from: https://reports.shell.com/sustainability-report/2019/servicepages/download-centre.html.

[99] Shell. Shell energy transition strategy 2021. 2021 [cited 1 May 2021]. Available from: https://www.shell.com/investors/annual-general-meeting/_jcr_content/par/textimage_d70a_copy.stream/1618407326759/7c3d5b317351891d2383b3e9f1e511997e516639/shell-energy-transition-strategy-2021.pdf.

[100] Shell. Shell Annual Report 2017 [cited 23 Jul 2020]. Available from: https://reports.shell.com/annual-report/2017/.

[101] Shell. Shell Annual Report 2018 [cited 23 Jul 2020]. Available from: https://reports.shell.com/annual-report/2018/.

[102] ExxonMobil. 2017 Sustainability Report Highlights. 2018 [cited 7 Feb 2020]. Available from: https://corporate.exxonmobil.com/-/media/Global/Files/sustainability-report/publication/2017-Sustainability-Report.pdf.

[103] ExxonMobil. Updated 2021 Energy & Carbon Summary. ExxonMobil 2021 [cited 17 May 2021]. Available from: https://corporate.exxonmobil.com/-/media/Global/Files/energy-and-carbon-summary/Energy-and-carbon-summary.pdf.

[104] BP. Sustainability Report 2017. 2018 [cited 7 Feb 2020]. Available from: https://www.bp.com/content/dam/bp/business-sites/en/global/corporate/pdfs/sustainability/group-reports/bp-sustainability-report-2017.pdf.

[105] BP. BP Sustainability Review 2011. 2012 [cited 6 Jan 2020]. Available from: https://www.bp.com/content/dam/bp/business-sites/en/global/corporate/pdfs/sustainability/archive/archived-reports-and-translations/2011/bp_sustainability_review_2011.pdf.

[106] Shell. Shell energy transition report [updated 2018; cited 16 Jul 2020]. Available from: https://www.shell.com/energy-and-innovation/the-energy-future/shell-energy-transition-report.html.

[107] ExxonMobil. 2015 Financial & Operating Review. 2016 [cited 21 May 2021]. Available from: https://corporate.exxonmobil.com/-/media/Global/Files/investor-relations/investor-relations-publications-archive/2015-FNO-new.pdf.

[108] Shell. 2016 Shell Annual Report and Form 20-F. Shell [updated 2017; cited 4 Jun 2019]. Available from: https://reports.shell.com/annual-report/2016/servicepages/download-centre.php.

[109] Christophers B. Big oil companies are driven by profit–they won’t turn green by themselves | Brett Christophers [updated 25 May 2021; cited 23 Jul 2021]. Available from: https://www.theguardian.com/commentisfree/2021/may/25/big-oil-companies-profit-green-renewables-fossil-fuels-net-zero.

[110] Chevron. 2019 Annual Report. 2020 [cited 22 Jun 2020]. Available from: https://www.chevron.com/-/media/chevron/annual-report/2019/documents/2019-Annual-Report.pdf.

[111] ExxonMobil. 2011 Summary Annual Report. 2012 [cited 9 May 2019]. Available from: http://library.nioc.ir/free-e-resources/Exxon%20Mobil/2011%20Summary%20Annual%20Report.pdf.

[112] House Committee on Oversight and Reform. Oversight Committee Launches Investigation of Fossil Fuel Industry Disinformation on Climate Crisis. 2021 [updated 17 Sep 2021; cited 13 Nov 2021]. Available from: https://oversight.house.gov/news/press-releases/oversight-committee-launches-investigation-of-fossil-fuel-industry.

[113] McGreal C. ExxonMobil lobbyists filmed saying oil giant’s support for carbon tax a PR ploy [updated 1 Jul 2021; cited 13 Nov 2021]. Available from: https://www.theguardian.com/us-news/2021/jun/30/exxonmobil-lobbyists-oil-giant-carbon-tax-pr-ploy.

[114] Lamb WF, Minx JC. The political economy of national climate policy: Architectures of constraint and a typology of countries. Energy Research & Social Science. 2020; 64:101429. doi: 10.1016/j.erss.2020.101429

[115] Tindall D, Stoddart MCJ, Dunlap Riley E. Climate change denial 2.0 was on full display at COP26, but there was also pushback. 2021 [updated 19 Nov 2021; cited 19 Nov 2021]. Available from: https://theconversation.com/climate-change-denial-2-0-was-on-full-display-at-cop26-but-there-was-also-pushback-171639.

[116] Butler D. Exxon lobbyist questions urgency of climate’s catastrophic risks. The Washington Post. 2021 Nov 24. Available from: https://www.washingtonpost.com/climate-environment/2021/11/24/exxon-global-warming-climate-skepticism/ [updated 2021 Nov 24; cited 2021 Nov 25].

[117] Lamb WF, Mattioli G, Levi S, Roberts JT, Capstick S, Creutzig F, et al. Discourses of climate delay. Glob Sustain. 2020; 3. doi: 10.1017/sus.2020.13

[118] Schmuck D, Matthes J, Naderer B. Misleading Consumers with Green Advertising? An Affect–Reason–Involvement Account of Greenwashing Effects in Environmental Advertising. Journal of Advertising. 2018; 47:127–45. doi: 10.1080/00913367.2018.1452652

[119] SUPREME COURT OF THE STATE OF NEW YORK. New York City filed a lawsuit in New York State Supreme Court against three oil and gas companies and American Petroleum Institute alleging that the defendants violated the City’s Consumer Protection Law. SUPREME COURT OF THE STATE OF NEW YORK 2021 [cited 1 Dec 2021]. Available from: https://www1.nyc.gov/assets/home/downloads/pdf/press-releases/2021/Earth-Day-Lawsuit.pdf.

[120] Sæverud IA, Skjærseth JB. Oil Companies and Climate Change: Inconsistencies between Strategy Formulation and Implementation. Global Environmental Politics. 2007; 7:42–62. doi: 10.1162/glep.2007.7.3.42

[121] Rowlands IH. Beauty and the Beast? BP’s and Exxon’s Positions on Global Climate Change. Environ Plann C Gov Policy. 2000; 18:339–54. doi: 10.1068/c9752

[122] Vormedal I, Gulbrandsen LH, Skjærseth JB. Big Oil and Climate Regulation: Business as Usual or a Changing Business. Global Environmental Politics. 2020; 20:143–66. doi: 10.1162/glep_a_00565

[123] van D. Halderen M, Bhatt M, Berens, Guido A. J. M., Brown T J., van B. M. Riel C. Managing Impressions in the Face of Rising Stakeholder Pressures: Examining Oil Companies’ Shifting Stances in the Climate Change Debate. J Bus Ethics. 2016; 133:567–82. doi: 10.1007/s10551-014-2400-8

[124] Grumbach JM. Polluting industries as climate protagonists: cap and trade and the problem of business preferences. Bus Polit. 2015; 17:633–59. doi: 10.1515/bap-2015-0012

[125] Bousso R. Special Report: BP gambles big on fast transition from oil to renewables. Reuters Media. 2021 Sep 21. Available from: https://www.reuters.com/business/sustainable-business/bp-gambles-big-fast-transition-oil-renewables-2021-09-20/ [updated 2021 Sep 21; cited 2021 Dec 20].

[126] Lieberman MB, Lee GK, Folta TB. Entry, exit, and the potential for resource redeployment. Strat Mgmt J. 2017; 38:526–44. doi: 10.1002/smj.2501

[127] Hansen GH, Steen M. Offshore oil and gas firms’ involvement in offshore wind: Technological frames and undercurrents. Environmental Innovation and Societal Transitions. 2015; 17:1–14. doi: 10.1016/j.eist.2015.05.001

[128] Christophers B. Fossilised Capital: Price and Profit in the Energy Transition. New Political Economy. 2022; 27:146–59. doi: 10.1080/13563467.2021.1926957

[129] Choquet P-L. Piercing the corporate veil: Towards a better assessment of the position of transnational oil and gas companies in the global carbon budget. The Anthropocene Review. 2019; 6:243–62. doi: 10.1177/2053019619865925

[130] Le Billon P, Kristoffersen B. Just cuts for fossil fuels? Supply-side carbon constraints and energy transition. Environ Plan A. 2020; 52:1072–92. doi: 10.1177/0308518X18816702

