# Supplementary material for: The clean energy claims of BP, Chevron, ExxonMobil and Shell: A mismatch between discourse, actions and investments
Source: PLoS One. 2022 Feb 16;17(2):e0263596. doi: 10.1371/journal.pone.0263596 (PMC8849545; doi:10.1371/journal.pone.0263596)
Supplement: S1 File — (PDF) [file pone.0263596.s001.pdf]

Supplementary A1 Discourse analysis (method and omitted terms)

The 39 search terms chosen for the keyword analysis in annual reports were chosen iteratively in a two-step procedure. First, after identifying them from grey literature from the oil industry (e.g. [1–6]) , we then verified their accuracy and suitability for use in annual reports by using the search function in Acrobat Reader. We rejected those terms turning up a large portion of non-relevant hits. For example, we rejected “diversify”, after discovering it is mostly used in the sense of reducing financial risk rather than reducing the share of fossil fuels. Specifically, we set a condition of 80% as the minimum threshold for the ratio of accurate hits returned by a search term. Second, in conducting the first step, we identified new terms in annual reports that were clustered around our initial search string. Again, these were then fine-tuned and iteratively tested for accuracy. Finally, in assembling the final list of search terms, we also set alternatives to account for linguistic variations, synonyms, and British and American spelling.

In conducting this procedure, we omitted the following terms from our keyword analysis, as shown in the table below:

Table Omitted search terms

Omitted search term	Reasons for exclusion
accountability	Not commonly used in an environmental sense.
biofeedstock, biomaterial	Not found in sampled documents chosen for testing search terms.
diversif*	Used mostly in the sense of reducing financial risk rather than reducing the share of fossil fuels in portfolio.
divest*	In most cases, not used to indicate the sale of an asset for the purposes of reducing the carbon intensity of energy portfolio.
electric vehicle	Double counts with electric.
environment*	Mostly used to refer to non-environmental aspects such as regulatory environment, economic environment, etc.
fuel cell	Not found in sampled documents chosen for testing search terms.
GHG	In most cases, searching with this term would result in a double count with "emission", which frequently appears with "GHG". Since other phases related to GHG (e.g. GHG reduction, GHG intensity), we calculate this term by "GHG" minus "GHG emission".
hydro electricity	Repeats with electricity.
mitigat*	In most cases, term not used to indicate the reduction of climate pollutants.
nitrogen dioxide*	Not a GHG.
pathway	Not found in sampled documents chosen for testing search terms. Also, in many cases, not used to indicate a low-carbon pathway.
scenario*	Most commonly used by Shell and BP. Searching with this term would thus generate results biased against Chevron and ExxonMobil. Also, even for the European pair, in many cases, use of "scenario" does not refer to low-carbon scenarios.
SDG	Not found in sampled documents chosen for testing search terms.
storage	Commonly used in the sense of storing hydrocarbon fuels.
sulfur dioxide*	Not a GHG.
synthetic	Mostly used to refer to conventional, carbon-based fuels and lubricants.
temperature	Often used in non-climate sense.
transform*	In most cases, not used to indicate the transition to clean energy.

References

[1] N. Pinko, K. Mulvey, B. Ekwurzel, P.C. Frumhoff, N. Hurd, J. Sideris, The 2018 Climate Accountability Scorecard: Insufficient Progress from Major Fossil Fuel Companies, 2019. www.ucsusa.org/climatescorecard (accessed 20 November 2019).

[2] K. Mulvey, J. Piepenburg, G. Goldman, P.C. Frumhoff, The Climate Accountability Scorecard: Ranking Major Fossil Fuel Companies on Climate Deception, Disclosure, and Action, 2017. https://www.ucsusa.org/resources/climate-accountability-scorecard (accessed 3 February 2019).

[3] J. Krane, Climate change and fossil fuel: An examination of risks for the energy industry and producer states, MRS energy sustain. 4 (2017). https://doi.org/10.1557/mre.2017.3.

[4] CTI, Breaking the Habit - Why none of the large oil companies are “Paris-aligned”, and what they need to do to get there - Carbon Tracker Initiative, 2019. https://carbontracker.org/reports/breaking-the-habit/ (accessed 1 March 2020).

[5] IEA, The Oil and Gas Industry in Energy Transitions: Insight from IEA analysis, 2020. https://www.iea.org/reports/the-oil-and-gas-industry-in-energy-transitions (accessed 25 October 2020).

[6] OGCI, OGCI Progress Report 2020, 2021. https://oilandgasclimateinitiative.com/wp-content/uploads/2020/12/OGCI-Progress-Report-2020.pdf (accessed 2 March 2021).
